# Impact of prophylactic hydroxychloroquine on ultrastructural impairment and cellular SARS-CoV-2 infection in different cells of bronchoalveolar lavage fluids of COVID-19 patients

**DOI:** 10.1038/s41598-023-39941-6

**Published:** 2023-08-05

**Authors:** Shikha Chaudhary, Arti Joshi, Kishore Sesham, Preeti Rai, Shailendra Kumar, Asit Ranjan Mridha, Upendra Baitha, Tapas Chandra Nag, Subhash Chandra Yadav

**Affiliations:** 1 Electron Microscope Facility, Department of Anatomy, All India Institute of Medical Sciences, New Delhi, Delhi 110029 India; 2 Department of Anaesthesiology, Pain Medicine and Critical Care, All India Institute of Medical Sciences, New Delhi, Delhi 110029 India; 3 Department of Pathology, All India Institute of Medical Sciences, New Delhi, Delhi 110029 India; 4 Department of Medicine, All India Institute of Medical Sciences, New Delhi, Delhi 110029 India

**Keywords:** Cell biology, Anatomy, Diseases, Pathogenesis, Signs and symptoms

## Abstract

Many drugs were recommended as antiviral agents for infection control and effective therapy to reduce the mortality rate for COVID-19 patients. Hydroxychloroquine (HCQ), an antimalarial drug, has been controversially recommended for prophylactic use in many countries, including India, to control SARS-CoV-2 infections. We have explored the effect of prophylactic HCQ from the cells of bronchoalveolar lavage fluids from COVID-19-induced acute respiratory distress syndrome patients to determine the level of infection and ultrastructural alterations in the ciliated epithelium, type II pneumocytes, alveolar macrophages, neutrophils, and enucleated granulocytes. Ultrastructural investigation of ciliated epithelium and type II pneumocytes showed lesser infections and cellular impairment in the prophylactic HCQ^+^ group than HCQ^−^ group. However, macrophages and neutrophils displayed similar infection and ultrastructural alterations in both patient groups. The enucleated fragments of granulocytes showed phagocytosis of the matured virus in HCQ^+^ groups. The present report unveils the ultrastructural proof to complement the paradox regarding the role of prophylactic HCQ in COVID-19 patients.

## Introduction

The COVID-19 outbreak caused by severe acute respiratory syndrome coronavirus-2 (SARS-CoV-2) has rapidly propagated with nearly half a billion infected human beings globally^1^. There were negligible reports on specific and effective treatments for this deadly infection. Due to sudden outbreaks and very high mortality (29%)^2^ by delta variant, many random trials and repurposing of existing drugs were conducted to control and cure the COVID-19 disease^2,3^. Hydroxychloroquine (HCQ), an antimalarial drug, has gained significant attention in the initial phase of COVID-19 from May 2020 onwards^4,5^. This drug was earlier reported to be effective (in vitro) in reducing viral internalization (by blocking proteolytic activation of S-protein) and replication (increasing the acidic environment of the endosome to inhibit viral assembly), including the SARS-CoV-2 and MERS-CoV^6–8^. The anti-SARS-CoV-2 effect of HCQ (by inhibiting internalization and proliferation) was proposed due to its ability to increase endosomal acidification, reduction of cathepsin L activation, interference with ACE-2 terminal glycosylation, proteolytic self-activation of furin, and the blockage of clathrin-mediated endocytosis^4,9–11^. The immunomodulatory effects, alkalinization of vacuolar pH, Zinc ionophores, and binding ability of HCQ to sialic acids were proposed to inhibit the COVID-19 infection in vitro non-specifically^9,12–14^.

Many clinical trials were initiated in various countries to investigate the effect of HCQ in the control and cure of COVID-19 disease^15–17^. It was reported that HCQ was very effective in reducing the multiplication of the SARS-COV-2 virus under in vitro culture conditions using Vero E6 cells with 6.90 µM concentration (EC)_90_^18^. This drug was prescribed in China at 500 mg twice daily for ten days for mild, moderate, and severe SARS-CoV-2 infection. Dutch center for disease control suggested 600 mg of chloroquine base (6 tablets A-CQ 100 mg) followed by 300 mg after 12 h on day 1, then 300 mg on days 2–5. To overcome the surge of COVID-19 in 2020, the Indian Council of Medical Research released an advisory to concede HCQ (400 mg twice on day one following the 400 mg dose once a week up to 3 to 7 weeks) as a prophylaxis dose before any symptoms arise to reduce the infection risk^19^. This type of oral dosing was reported to achieve a favorable pharmacological concentration in vivo due to the very effective pharmacology of this drug^20–22^.

Hydroxy chloroquine (HCQ), a chloroquine derivative, was found more effective because of its better water solubility, low toxicity, and more prolonged circulation^5,8,18,23^. A randomized clinical trial by Pujol et al. shows the safety of HCQ in the low-level dosage^24^. The study by Serrano et al. reported the efficiency of HCQ in minimizing viral infectivity to some extent^25^. Considering many similar pharmacological findings, this drug was offensively introduced as a prophylactic agent to control the SARS-CoV-2 viral infection despite associated side effects. However, the negligible impact of HCQ on COVID-19 was reported using calu-3 cell lines (lung adenocarcinoma) and in vivo cynomolgus macaque in the late months of the year 2020^11,26^. Several studies have disclosed doubt about HCQ action in controlling the COVID-19 infection^27^. Due to insignificant mortality reduction in admitted COVID-19 patients, WHO (world health organization) had also announced the insufficiency of the HCQ in the treatment of COVID disease^28^. However, the dissatisfaction with HCQ efficiency by the number of clinical trials studies should not be taken as the non-effectiveness of this drug as a broad-spectrum antiviral agent. The study by Pandolfi et al. on cellular and variable cytokines levels in the BALF implies that the anti-SARS-CoV-2 effect of HCQ drug could be explored through an ultrastructural examination of HCQ-treated patients^29,30^. Several reports published in mid-2020 (March to October 2020) supported the positive effects of HCQ in controlling the infection and multiplication of the SARS-CoV-2 virus^4,16,31–36^. Liu et al. demonstrated in vitro that HCQ blocks the transport of SARS-CoV-2 from early endosomes to endo-lysosomes resulting in an abnormally enlarged, more significant number of early endosomal vesicle^4^. A study done by Ruiz et al. (2021) revealed the presence of a pharmacological concentration of HCQ in the fluid of epithelial lining and the lung (higher than epithelium) of intubated COVID-19 patients^37^. However, many editorial and initial reports suspected the efficacy of HCQ in COVID-19. They warned about the adverse effect of indiscriminate usage supported by research showing the ineffectiveness of the HCQ in the control or cure of COVID-19^8,38–42^. Several clinical trials showed an insignificant effect of HCQ in COVID-19 patients^27^. Considering the controversial report on the impact of HCQ on COVID-19 under in vitro and in vivo conditions, this study was designed to explore the effect of HCQ on the ultrastructural level of the various cells from the bronchoalveolar lavage fluids (BALF) of severely infected and intubated COVID-19 patients. We have compared the ultrastructures of ciliated epithelium, type II pneumocytes, alveolar macrophage, neutrophils, and enucleated granulocytes from the BALF of the mildly infected non-ARDS (intubated due to trauma condition, HCQ^−^), severe ARDS patients with and without prophylactic HCQ.

## Results

**Figure 1:**
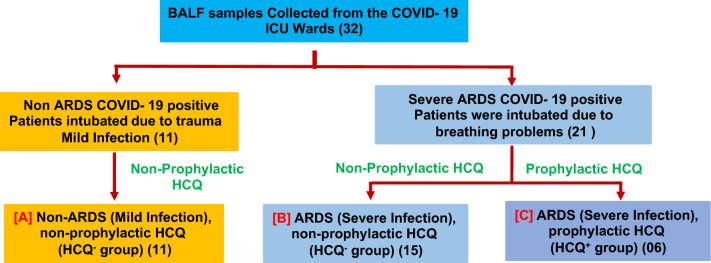
Study design to evaluate the effect of prophylactic HCQ on the ultrastructure of various BALF cells. The BALF samples (32 samples) were collected from the intubated patients. The patients were finally grouped under the (**a**) Non-ARDS, HCQ^−^ group (**b**) ARDS, HCQ^−^ group and (**c**) ARDS, HCQ^+^ group. The mild COVID-19 patients (A) were intubated due to traumatic condition (not due to COVID-19 induced ARDS) but found COVID-19 positive through RT-PCR. The BALF of these patients were taken to explore the ultrastructural changes between the prophylactic HCQ^+^ and HCQ^−^ groups. The three groups were selected to differentiate the effect of the prophylactic HCQ on the ultrastructural changes in ciliated epithelium, type 2 pneumocytes, alveolar macrophage, neutrophils, and non-nucleated cytoplasmic fragments) of the BALF. The number of patients recruited in this study was indicated along with each subgroup.

The effect of prophylactically taken HCQ (taken by the healthy individual before SARS-CoV-2 infection, HCQ^+^) on different cells from the BALF of severe ARDS (intubated) patients was evaluated on the level of infection and ultrastructural alterations. These ultrastructural findings were compared with the severe ARDS patients who have not taken HCQ as a prophylactic dose (HCQ^−^). Patients who had taken prophylactic HCQ and later developed SARS-CoV-2-induced ARDS were recruited for this study (Supplementary Tables S2 & S3). All the ARDS patients who were given HCQ after the infection were excluded from the study. Mild COVID-19 patients (non-ARDS) were also recruited in this study to evaluate the impact of SARS-CoV-2-induced ARDS on the level of infection and ultrastructural modulation of BALF cells. These patients were intubated due to trauma and subsequently infected with SARS-CoV-2, showing mild clinical symptoms (Fig. 1). The effects of HCQ on the ultrastructure of ciliated epithelium, type II pneumocytes, macrophages, neutrophils, and enucleated granulocytes cells were examined in this study.

### Effect of HCQ on ciliated epithelium from the BALF

**Figure 2:**
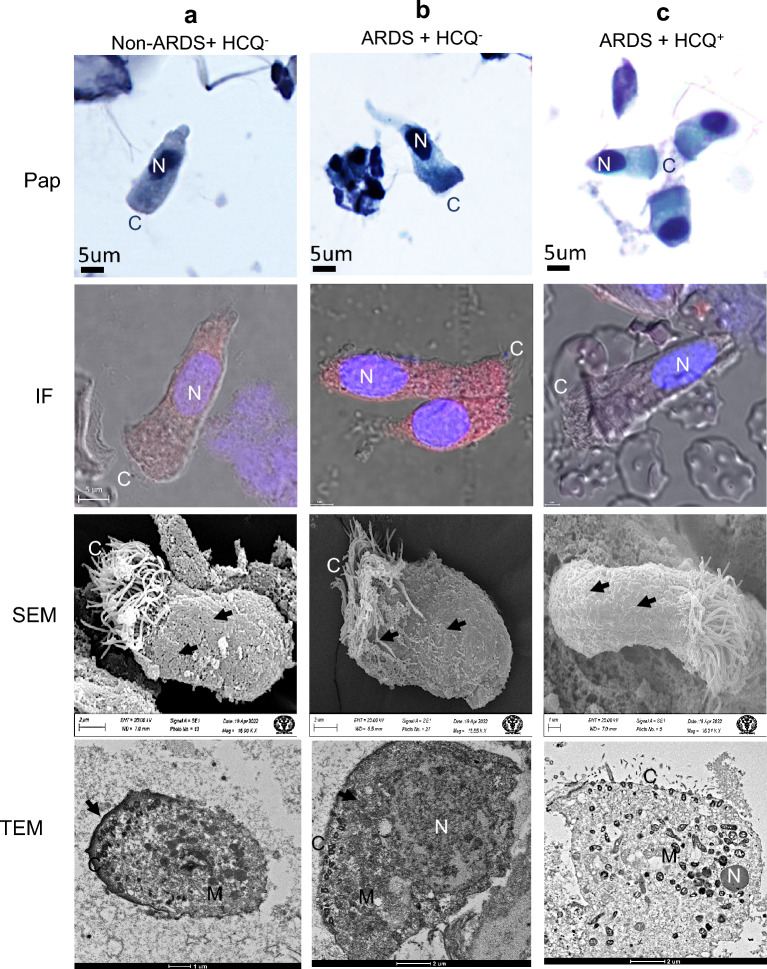
Ciliated epithelium from the BALF of intubated COVID-19 patient. (**a**) Mild infection (non-ARDS) HCQ^−^ group (**b**) Severe infection (ARDS) HCQ^−^ and (**c**) Severe infection (ARDS) with prophylactic HCQ^+^ group. Ciliated epithelium of each group of patients was imaged using the PAP test for identification of various cells in BALF (PAP), Immunofluorescence (merged image of DIC, DAPI, and Alexa flor 598) imaging using SARS-CoV-2 virus S protein-specific antibody for the determination of the level of infection through Fiji software (IF), Scanning electron microscopy for surface imaging of virus and to visualize the cellular morphology (SEM), and cellular ultrastructural details using transmission electron microscopy (TEM). The level of infections was highest in patient group B, followed by groups A and C. The cilia were mostly intact in each group of patients. SARS-CoV-2-like virus particles were seen on the surface and in the cell membrane (arrows). The ultrastructural damage was more prominent in patient group b, followed by groups c and a. *N* Nucleus, *C* Cilia, *M* Mitochondria, *arrows* SARS-CoV-2 virus.

The ciliated epithelial cells from the BALF of mild infection (non-ARDS, HCQ^−^) patients showed intact cellular structure, vacuolated cytoplasm with a dense nucleus (in PAP imaging), and a mild cellular infection (Table 1) under immunofluorescence (IF) imaging (Fig. 2a). The surface ultrastructure under SEM imaging showed many virus-like structures in the body of cells and cilia (Fig. 2a). TEM imaging of these cells exhibited healthy mitochondria, many basal ciliary complexes (identifiable features for ciliated cells), and some virus-like structures (< 100 nm) on the plasma membrane and in peripheral cytoplasm. We could not find any virus-like particles containing membrane-bound vesicles in these cells (Fig. 2a). These cells from the ARDS patients without HCQ (HCQ^−^)showed a highly vacuolated cytoplasm (indicative of higher viral load), with a dense nucleus under light microscopy and higher immunofluorescence (high infection) (Fig. 2b). A scanning electron microscope showed skeletal cellular morphology with many virus-like particles on the body and cilia of the cells (Fig. 2b). TEM revealed many membrane-bound vesicles with granular virus-like particles (arrow). Many ciliary basal bodies were seen as identifiable features of ciliary cells. The disappearance of the plasma membrane and swollen mitochondria showed typical initial apoptotic-like characteristics indicative of higher viral infection (Fig. 2b).

**Table 1:** Fluorescence intensity determination using Fiji software for the many cells in each patient group (from IF study).

Type of cells	Patient group	Fluor. intensity mean ± SD	No of cells analyzed
Ciliated epithelium	A	17.71 ± 4.10	12
	B	22.02 ± 5.32	10
	C	12.17 ± 2.69	14
Type II pneumocytes	A	19.53 ± 3.67	18
	B	19.99 ± 2.49	10
	C	11.73 ± 1.73	09
Macrophages	A	14.47 ± 3.30	17
	B	15.29 ± 2.50	11
	C	15.33 ± 2.39	08
Neutrophils	A	15.55 ± 3.73	14
	B	15.51 ± 2.73	13
	C	15.41 ± 2.83	09
Anucleated granulocytes	A	23.74 ± 3.09	07
	B	23.79 ± 3.07	04
	C	23.49 ± 3.06	05

The severe ARDS patients with prophylactic HCQ (HCQ^+^) showed relatively healthy ciliated epithelial cells in PAP imaging with active cytoplasm and normal nucleus with the nucleolus. These cells showed significantly less immunofluorescence, indicative of the mild infection in these cells. The surface ultrastructure of these cells showed a healthy appearance of the cells with very few virus-like particles on the plasma membrane and cilia. Transmission electron microscope imaging showed healthy mitochondria, dense cytoplasm, eccentric nucleus, negligible membrane-bound vesicles, many virus-like structures on the outer surface of the plasma membrane, and typical vacuoles (Fig. 2c).

### Effect of HCQs on type II pneumocytes

**Figure 3:**
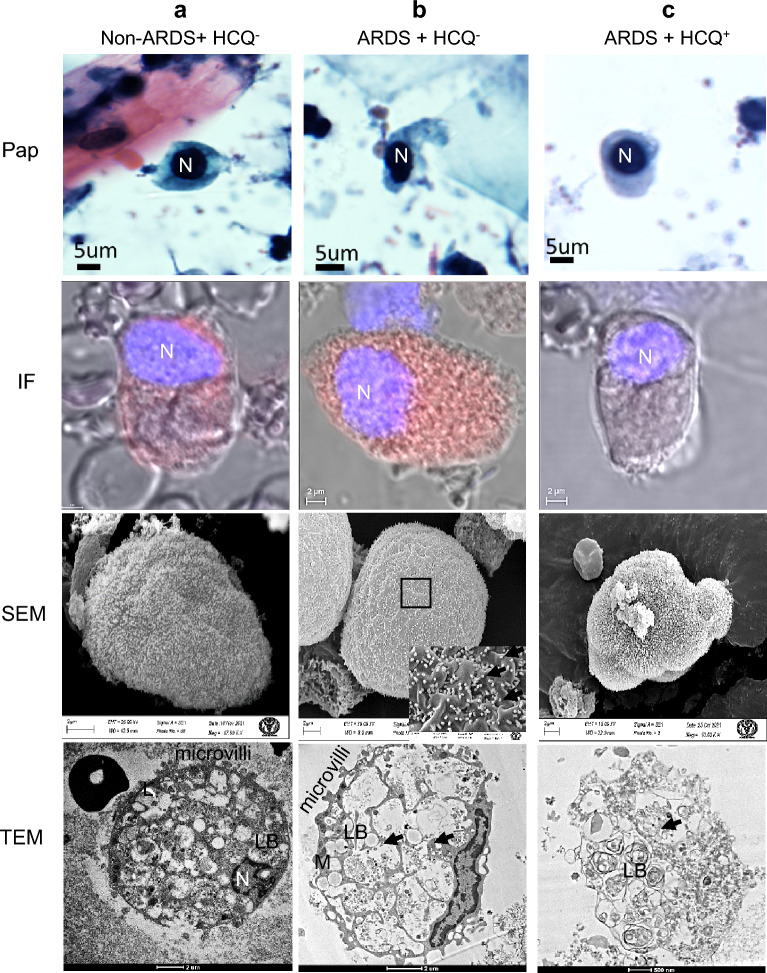
Type II pneumocytes from the BALF of intubated COVID-19 patients. (**a**) Mild infection (non-ARDS) HCQ^−^ group (**b**) Severe infection (ARDS) without HCQ (HCQ^−^) and (**c**) Severe infection (ARDS) with prophylactic HCQ (HCQ^+^) group. These cells showed a membrane projection, a characteristic feature of type II pneumocytes. The infection was highest in group B patients, followed by groups A and C. This indicates the effectiveness of HCQ in protecting the infection in these cells from the SARS-CoV-2 virus. SEM imaging confirms the presence of a microvilli-like structure along with virus-like particles on the surface (arrow). The characteristics of the lamellar body were intact and had clear, distinguishable morphology in group C; however, these were released in group B patients. *N* Nucleus, *M* Mitochondria, *L* Lipid body, *LB* Lamellar body, *arrows* SARS-CoV-2 virus.

Type II pneumocytes of mild COVID-19 patients (non-ARDS, HCQ^−^) exhibited a dense nucleus with vacuolated cytoplasm and mild immunofluorescence. However, the surface ultra-structures of these cells were covered with typical multiple microvilli-like morphologies, which suggest healthy cells (Fig. 3a). TEM imaging revealed the eccentric nucleus, with many intact surfactant granules, lipid body, healthy mitochondria, and residual bodies. The presence of many ferritin granules indicates the oxidative stress condition of the cells at the mild infection stage. Many virus-like particles were present on the plasma membrane of these cells and in cytoplasmic projections (Fig. 3a). However, type II pneumocytes altered their morphology in severe ARDS conditions (HCQ^−^). It shows moderate to high immunofluorescence as a sign of higher infection (Table 1). SEM revealed the swollen morphology, loss of curvatures, reduction in microvilli, and cell size enlargement. TEM showed a complete disintegrated nucleus, many membrane-bound vesicles, plenty of virus-like particles in the membrane-bound vesicles, vacuolized cytoplasm, lean lipid body, and many released surfactant granules. All these findings indicate the high stress and initial apoptotic condition of these cells under the influence of high SARS-CoV-2 infections (Fig. 3b).

However, the patient in the prophylactic HCQ^+^ group presented healthier cells and nucleus in optical microscope imaging, with significantly less immunofluorescence. Minor infection may be due to the lesser internalization and multiplication of the SARS-CoV-2 under the influence of HCQ. These cells from the prophylactic HCQ^+^ patients showed normal surface morphology under SEM imaging. However, some cells showed necrosis with plenty of surfactant granules under TEM imaging (Fig. 3c). Many cells showed intact morphology with a stressed nucleus but intact surfactant granules.

### Effect of HCQs on alveolar macrophages

**Figure 4:**
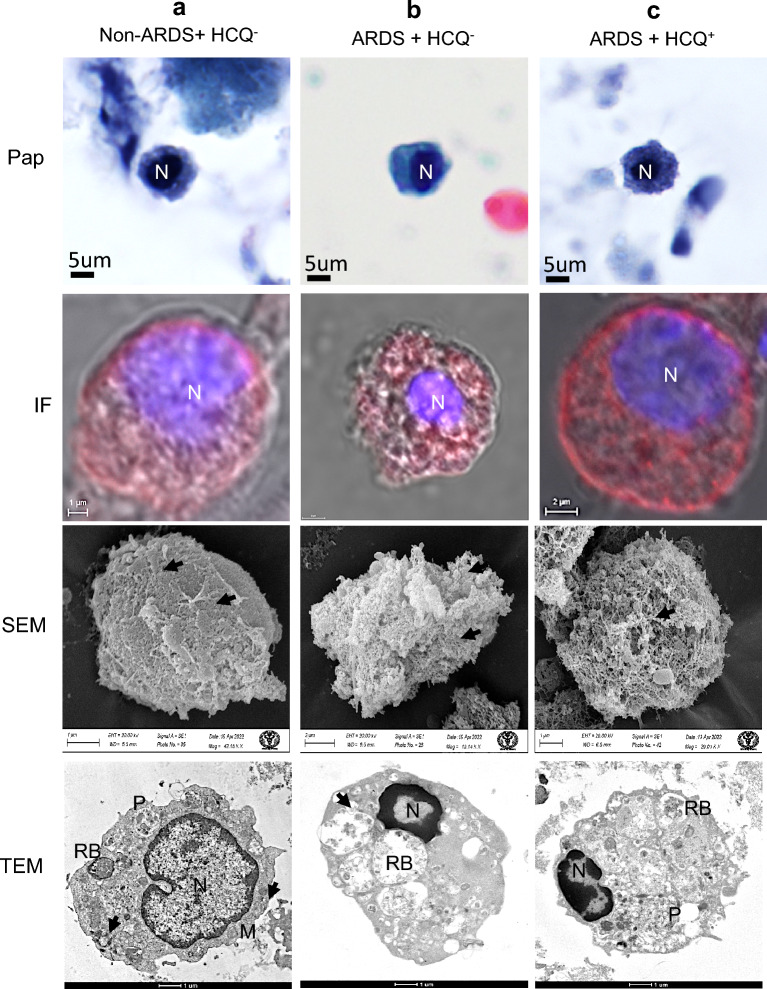
Macrophages/monocytes from the BALF of intubated COVID-19 patients. (**a**) Mild infection (non-ARDS) and HCQ^−^ group (**b**) Severe infection (ARDS) patients without HCQ (HCQ^−^) and (**c**) Severe infection (ARDS) with prophylactic HCQ (HCQ^+^) group. The IF study showed a severe infection in all the patient groups, indicating the ineffective role of HCQ in the alveolar macrophage. SEM images showed multiple virus-like structures on the cell surface (arrows) with similar surface morphology in ARDS patients (**b**, **c**). TEM images reveal phagosomes on the cell cytoplasm with many viruses (arrows). There was similar ultrastructural damage in ARDS patient groups (**b**, **c**). The non-ARDS group (**a**) showed intact cellular ultrastructure even with higher infection. *N* Nucleus, *M* Mitochondria, *RB* Residual Body, *P* Phagosome, *arrows* SARS-CoV-2 virus.

To identify and compare the ultrastructural alterations in alveolar macrophage of mild, severe ARDS (HCQ^−^) and prophylactic HCQ^+^ patients were imaged using PAP, IF, SEM and TEM to observe the number of surface projections, ultrastructure, and cytoplasm along with the presence of virus-like particles. The macrophage of mild infection patients showed relatively vascular cytoplasm and dense nucleus with moderate to heavy infections (Table 1). The surface morphology by SEM imaging showed a typical rough surface with the presence of very few virus-like structures. TEM revealed a horseshoe-shaped nucleus with peripheral euchromatin, nucleolus, dense cytoplasm, multiple phagosomes, and less cytoplasmic projection indicative of healthy cells (Fig. 4a). The ARDS patients (HCQ^−^) showed proliferative alveolar macrophage cells with very high immunofluorescence. The surface morphology showed the typical hyperactive macrophage with plenty of viruses on the surface. These cells showed an initial apoptotic nucleus with large size phagosomes filled with virus-like particles (arrow). This specifies the highly proliferative condition of these cells under the influence of cytokine storms (Fig. 4b). This macrophage from the prophylactic HCQ^+^ patients showed moderate infection and proliferative surface morphology. However, TEM revealed a healthy nucleus and cells with plenty of filled phagosomes. The ultrastructural alteration was similar to that of the HCQ^−^ group (Fig. 4c).

### Effect of HCQs on neutrophils

**Figure 5:**
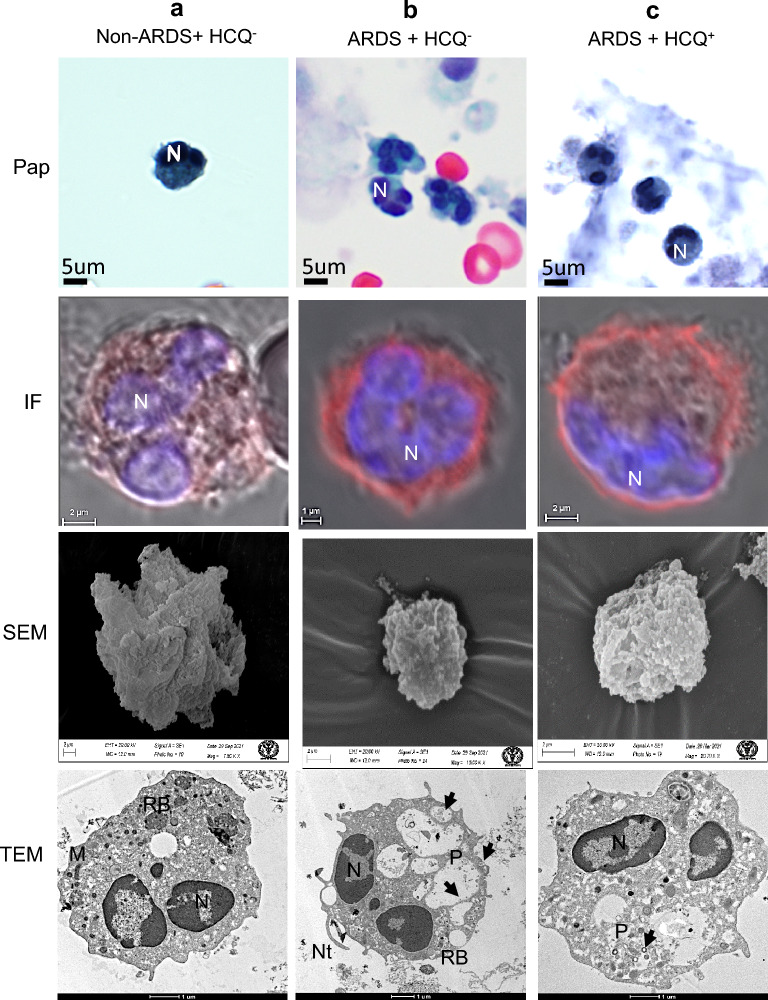
Neutrophils from the BALF of intubated COVID-19 patient. (**a**) Mild infection (non-ARDS) HCQ^−^ group (**b**) Severe infection (ARDS) patients without HCQ (HCQ^−^) and (**c**) Severe infection (ARDS) with prophylactic HCQ (HCQ^+^) group. Multilobed disintegrating nucleus with external NETs was observed in all the patient's subgroups. The level of infection (IF) was significant in ARDS groups (**b**) and (**c**) and mild in group (**a**). HCQ has no effects on neutrophil cells for the control of infection and virus internalization. SEM images showed multiple virus-like structures on the cell surface. TEM images reveal phagosomes on the cell cytoplasm with many viruses (arrows). HCQ did not show a protective effect on the infection of these cells. *N* Nucleus, *M* Mitochondria, *RB* Residual Body, *P* Phagosome, *Nt* neutrophil extracellular traps, *arrows* SARS-CoV-2 virus.

Neutrophils from the BALF sample of mild infectious patients showed vascular cytoplasm with a multi-lobed nucleus with mild immunofluorescence. The surface morphology showed a shrunken cellular body under SEM imaging. Their nuclei exhibited stress-like conditions with a peripheral accumulation of the heterochromatin and centrally located euchromatin. The presence of multiple and scattered healthy mitochondria with other cytoplasmic organelles confirms the activated neutrophils without neutrophils extracellular traps (NETs) (Fig. 5a). However, dense nuclei and condensed cytoplasms under light microscopy from the severe ARDS patients confirmed the high proliferative condition of these cells with many NETs-like structures. These cells also showed relatively higher immunofluorescence, suggestive of severe infection. The surface ultrastructure showed many NETs-like structures designating the high-stress conditions in these cells. TEM revealed a more heterochromatic nucleus, indicating the initial apoptotic condition with many vacuoles having virus-like structures and many NETs. The fewer mitochondria with many viruses on the cell surface confirm the initial apoptotic condition caused by SARS-CoV2 viral infection (Fig. 5b).

Neutrophils from the severe ARDS patients with prophylactically taken HCQ^+^ group displayed better cellular and nuclear morphology with relatively moderate infections, as indicated by the immunofluorescence (Table 1). However, the surface morphology revealed plenty of small NETs like projections. These NETs' projections were also appreciated in the transmission electron microscope imaging with plenty of vacuoles containing immature virus-like particles, heterochromatin-rich nuclei, and many small mitochondria. These neutrophils showed a similar proliferative condition to that of HCQ^−^ group (Fig. 5c).

### Enucleated granulocytes

**Figure 6:**
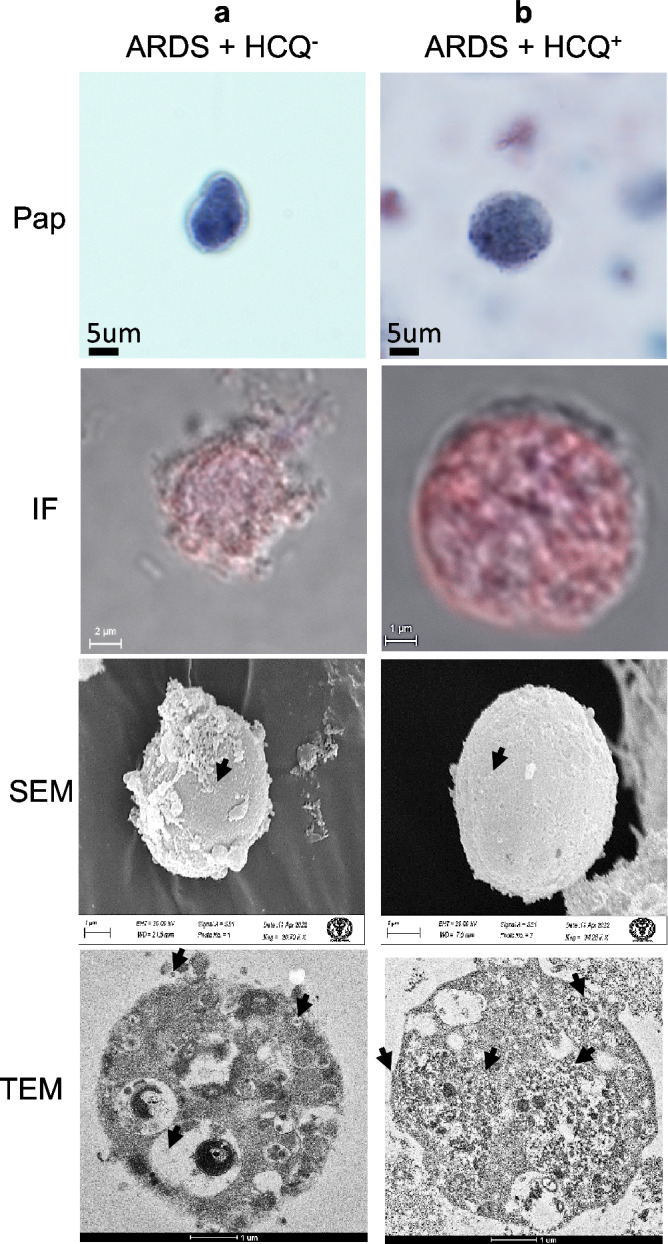
Enucleated granulocytes from the BALF of intubated COVID-19 patient. (**a**) ARDS patients without HCQ (HCQ^−^) and (**b**) ARDS with prophylactic HCQ (HCQ^+^) group. Many non-nucleated cells were observed in the BALF, having the highest level of SARS-CoV-2 infection in comparison to other cells. These cells showed granulocytes origin showing CD15 positive. TEM imaging showed highly vacuolated phagosomes in both groups. However, the HCQ-treated group cells showed entirely filled phagosomes with mature viruses. *Arrows* SARS-CoV-2 virus.

BALF contains many enucleated cells derived from the granulocytes of the blood after the degradation of the nucleus under the influence of a higher infection of SARS-CoV-2. These cytoplasmic structures showed intense fluorescence, suggesting high viral loads. We could not find enucleated granulocytes in the mild infection patients. The ARDS patients showed high immunofluorescence with NETs-like structures on the surface. TEM revealed plenty of viruses in the membrane-bound vesicles without the nucleus. We have sectioned and imaged these cells entirely but could not locate the presence of a nucleus by TEM. Some peripheral vacuoles showed the virus's release on the cells' surface (Fig. 6a). The patients with prophylactic HCQ showed granular cytoplasm, moderated to severe infection as signified by high immunofluorescence. The surface morphology was a typical rounded structure with plenty of mature virus-like particles. TEM exhibited eccentrically tiny fragments of slight nuclear residue, with plenty of vesicles, phagosomes, and small virus particles, confirming the SARS-CoV-2 virus. The HCQ^+^ group cells showed very high phagocytosis of the mature SARS-CoV-2 virus (Fig. 6b).

## Discussion

In this comparative study, the effects of HCQ on different cells found in the BALF of COVID-19 patients with respect to the level of SARS-CoV-2 viral infection, impact on the cell surface, and other cell organelles (ultrastructure) were evaluated by light and electron microscopy. This study was done on the BALF samples of COVID-19-positive intubated patients to understand the effect of HCQ^+^ versus HCQ^−^ by ultrastructural comparison and level of infection. BALF proved to be a valuable sample for studying the SARS-CoV-2 virus proliferation and the effects of prophylactic HCQ on COVID-19. The electron microscopical examination of the SARS-CoV-2-infected cells was performed to evaluate the impact of HCQ on the ultrastructural changes and viral spread in severe ARDS patients. Ciliated epithelium cells of prophylactic HCQ^+^ patients showed a significantly lower infection (immunofluorescence) and healthy nucleus/cytoplasm than the HCQ^−^ group (Fig. 2). This specifies that prophylactic HCQ plays a preventive role in the internalization of the SARS-CoV-2 virus in ciliated epithelium. Relatively fewer virions in characteristic membrane-bound vesicles, viral lining on the cell surface, and a lower number of double-membrane vesicles in HCQ^+^ patients provide clear evidence of the reduction of SARS-CoV-2 infection (Fig. 2). This also suggest the reduction of replication of viral particles in the ciliated epithelium of the HCQ^+^ group during the late phase of the infection. SARS-CoV-2-infected cells usually show an irregular morphology with spreading from the edge to the center of the cell layer. These infected cells also showed a gradual appearance of small aggregates, and increased interstitial particles. The diseased cells became rounded, condensed, detached, or fused^43^. In our study, the HCQ^+^ cells showed relatively lesser changes which indirectly proves the impact of HCQ on the control of infection in these cells at the ultrastructural level (Fig. 2).

Type II pneumocytes cells release surfactants, which play a significant role in immune defense, and airway regeneration during lung injury^44^. These cells from the HCQ^+^ group showed relatively healthy cytoplasm with lesser infection than mild and ARDS patients without prophylactic HCQ (HCQ^−^ group). This provides direct evidence of the infection control by the prophylactic HCQ^+^ in these cells. It was already reported that Type II pneumocytes have a higher level of HCQ in the prophylactic group of patients than any other cells of the lungs^37^. A significantly lower number of characteristic hemilamellar bodies (generated due to viral infection) were observed among these cells under electron microscopy compared to severe ARDS patients (Fig. 3). These organelles were filled with unequal numbers of mature virions in the markedly expanded rough endoplasmic reticulum and vesicles. The viral particles in these cells were observed as circular objects in the vesicles, which may be poly-vacuolar bodies or autophagic vesicles that engulfed particles. TEM imaging of these cells showed that SARS-CoV-2 internalized in cells through membrane fusion and matured in vesicles (Fig. 3).

Histopathological analysis of BALF of ARDS patients revealed fibroproliferative responses of alveolar macrophages with diffuse alveolar damage in all the groups of patients. The negligible ultrastructure changes in alveolar macrophages of mild infection patients may be due to a low level of infection. The ultrastructures revealed that these macrophages were not activated. Monocytes and macrophage activation have been reported as the primary cause of ARDS-like inflammatory syndrome by expressing 2 to 3 times more inflammatory cytokines^45,46^. However, in severe ARDS patients, the foamy appearance indicates the hyperactive and apoptotic macrophage. This is due to various sizes of vesicles and fibrin accumulation in the vicinity of cell membrane. Similar morphology of these macrophages in the HCQ^+^ group revealed no effect of the HCQ in the cellular ultrastructure (Fig. 4).

The viral replication inside the cells alarms a cellular injury that leads to the second line of the innate response by releasing inflammatory mediators. Due to inflammation, circulating neutrophils and monocytes rush to the site of infection, where they start phagocytosis of the invading virus. The normal cellular ultrastructure of neutrophils of the mild infection patients group indicated less inflammation. However, an apparent cytopathic effect was observed in the severe and HCQ^+^ patients group showing no infection protection effect of HCQ on these cells. The vacuolar cytoplasm with plenty of virus-like structure and NETs were indicators of activated neutrophils (Fig. 5).

Multiple anucleate cells or their fragments with a very high level of infection in severe ARDS patients with and without HCQ indicate the negligible impact of HCQs in these cells. These cells have identified a granulocyte due to CD 15 positive in the immunohistochemistry^47^. The phagocytic vesicles were filled with mature viruses indicating that HCQs may increase the phagocytic activity of granulocytes (Fig. 6).

## Conclusions

This study highlighted the level of SARS-CoV-2 infection and ultrastructural alteration in the prophylactic HCQ^+^ group in comparison to the HCQ^−^ group of the ciliated epithelium, type II pneumocytes, alveolar macrophage, neutrophil and anucleated granulocytes individually. We found the significant antiviral activity of HCQ as a protective role rather than a degenerating effect on the ciliated epithelium and type II pneumocytes in which low infection level and relatively intact cellular ultrastructure were observed. The ultrastructure of alveolar macrophages and neutrophils were degenerated in ARDS patients of both the HCQ^+^ and HCQ^−^ group. However, enucleated fragments of granulocytes showed a higher tendency of phagocytosis of the mature SARS-COV-2 virus in the HCQ^+^ group.

## Methods

### Material

BSA and ethanol were procured from Himedia. Triton X-100 was procured from Fisher Scientific. Osmium tetroxide was procured from Ted Pella, USA. Uranyl acetate was from TAAB, UK, and lead citrate from Ladd. Polyclonal anti-SARS-CoV-2 specific primary antibody (Cat no. ab275759) and Alexa fluor-594 conjugated anti-rabbit secondary antibody (Cat no. ab150080) were procured from Abcam, Plc, UK. Karnovsky’s fixative (0.5% glutaraldehyde + 2.0% paraformaldehyde), hematoxylin, eosin, orange G, Scott’s water, xylene, DPX, PBS, poly-l-lysine, epoxy embedding kit, and DAPI were purchased from Sigma chemical company, MO, USA.

### Ethics statement

BALF from the mild and severe SARS-CoV-2 infected (COVID-19 positive) and intubated patients from the Intensive Care Unit (ICU) were collected after taking informed consent from all participants or patient representatives. The study was approved by Institutional Ethics Committee (IEC), All India Institute of Medical Sciences New Delhi, India (Ref. No. IEC-307/27.04.2020, RP-10/202). We are confirming that all experiments were performed in accordance with relevant guidelines and regulations.

### Study design and sample collection

BALF samples were collected from intubated SARS-CoV-2 positive patients in the COVID-19 intensive care unit (ICU), AIIMS, New Delhi. All samples were collected between 3rd October 2020 and 31st January 2021 (Supplementary Table S1). The patients were categorized into three groups such as (A) mild infection non-ARDS patients (non-ARDS, HCQ^−^, 11 patients), (B) severe infection with ARDS without prophylactic HCQS (ARDS, HCQ^−^, 15 patients), and (C) Severe ARDS patients that had taken HCQS before the SARS-CoV-2 infection as prophylactic dose (ARDS, HCQ^+^, 06 patients) (Fig. 1). RT-PCR test was performed to confirm the COVID-19 infection for all the patients recruited in the study. The patients who had taken HCQ after the COVID-19 symptoms were excluded from the study. The ARDS patients were intubated due to hypoxemia (SpO_2_ < 90%), high oxygen requirement (flow rate 20–25 L/min), and deteriorating breathing problems.

The BALF (15–20 mL) was primarily fixed in freshly prepared 20 mL, 2X Karnovsky’s solution (final 5% glutaraldehyde + 4.0% formaldehyde) in 0.2 M phosphate buffer. The surface of sample vials was sterilized by alcohol/soap solution by incubating for two hours at room temperature and stored at 4 °C in a COVID-19 designated refrigerator. The medical records of all patients were reviewed and cross-checked by an on-duty medical physician.

### Sample processing for the cellular enrichment

After the primary fixation, the BALF solution was diluted ten times with 0.1 M NaCl solution and strained through a nylon mesh cell strainer with a 100 µm pore. The filtrate was centrifuged at 2500 rpm for 3 min in a swinging bucket. The cell pellets were washed 2–3 times for 10 min with PBS solution to remove the excess mucus. The cellular content was enriched by centrifugation at 1200×*g* for 3 min and resuspended again in the primary fixative A (0.5% glutaraldehyde and 2.0% paraformaldehyde in 0.1 M PB buffer). These samples were processed for PAP staining, immunofluorescence (IF), scanning- and transmission electron microscopy (SEM, TEM).

### Immunofluorescence using SARS-CoV-2 spike protein-specific antibody

BALF was washed three times with 0.1 M phosphate buffer, and smears were prepared using 10 µL of sample on poly-l-lysine coated glass slides and air-dried at room temperature (RT). Smears were permeabilized by PBST (0.1% Triton X-100 in 1 × phosphate buffer saline, PBS) and after protein blocking with 2% BSA in PBS for 30 min, incubated with primary antibody (Abcam ab275759, polyclonal against S1 spike protein, dilution 1:500) for four hours in a humid chamber at room temperature. After washing with PBS fluorophore-conjugated secondary antibody (Alexa fluor-594 conjugated anti-rabbit secondary antibody, Abcam, Cat No.-150080; dilution 1:500;) was added for 1 h at RT in the darkroom. Smears were washed with phosphate buffer, and DAPI (1 μg/mL) was added for 5 min. Excess DAPI was washed with PBS, and smears were mounted with 90% glycerol. Fluorescence imaging was performed on a laser scanning confocal microscope (Leica SP8 Germany).

### Scanning electron microscopy

For SEM, the enriched and primary fixed cellular components of BALF were osmicated, dehydrated with ethanol, critical point dried (E-3100, Quorum Tech), and mounted on double-sided tape on the aluminum stubs. These stubs were sputter-coated with a gold-based sputter coater (HHV BT-150) for 180 s. Electron micrographs were obtained on EVO18 (Zeiss, Germany) SEM operated at 20 kV accelerating voltage, between 8 and 10 mm average working distance with SE detector, and magnifications ranging from 5000 × to 30,000 ×.

### Transmission electron microscopy

To prepare the specimens for TEM imaging, enriched cellular constituents of BALF were primarily fixed using 2.5% glutaraldehyde + 2.0% paraformaldehyde in 0.1 M phosphate buffer (PB). The fixed cellular pellets were washed with 0.1 M PB (pH 7.4) and post-fixed with 1% osmium tetroxide in 0.1 M PB (pH 7.4) for one hour (secondary fixation) at 4 °C. For two hours, pellets were washed with distilled water, and en bloc staining was done with 2% uranyl acetate in 50% ethanol. These samples were again washed with distilled water and dehydrated in an ethanol series (50%, 70%, 80%, 90%, and 100%). These pellets were infiltrated with toluene/resin and finally embedded in Araldite CY212 resin. The blocks were polymerized at 65 °C for 48 h. Resin blocks were trimmed, and 70 nm thin sections were prepared using UC7 ultramicrotome (Leica). The sections were mounted on grids and stained with 5% uranyl acetate and 5% lead citrate. Cells were imaged using Talos F200 Transmission Electron Microscope (Thermo Fisher Scientific) using a FEG filament operated at 200 kV.

### Supplementary Information

Supplementary Table S1.

Supplementary Table S2.

Supplementary Table S3.

Supplementary Information 1.

## Data Availability

The datasets (Microscopic images of PAP, IF, SEM, and TEM) used and/or analyzed during the current study are available from the corresponding author upon reasonable request.
