# Supplementary material for: Impact of prophylactic hydroxychloroquine on ultrastructural impairment and cellular SARS-CoV-2 infection in different cells of bronchoalveolar lavage fluids of COVID-19 patients
Source: Sci Rep. 2023 Aug 5;13:12733. doi: 10.1038/s41598-023-39941-6 (PMC10404249; doi:10.1038/s41598-023-39941-6)
Supplement: Supplementary file 1 — Supplementary Table S1. [file 41598_2023_39941_MOESM1_ESM.docx]

**Supplementary Table S1: Detail of the recruited patients in this study**

| **S. No** | **Patient groups** | **Age** | **G** | **Prophylactic HCQ taken** | **Patient Conditions** | **Covid -positive Date** | **Sample Collection Date** |
| --- | --- | --- | --- | --- | --- | --- | --- |
| 1 | A | 73 | M | No | Non-ARDS | 2020.12.30 | 2021.01.10 |
| 2 | A | 52 | F | No | Non-ARDS | 2021.01.30 | 2021.02.12 |
| 3 | A | 55 | F | No | Non-ARDS | 2020.12.30 | 2021.01.07 |
| 4 | A | 62 | F | No | Non-ARDS | 2021.02.06 | 2021.02.14 |
| 5 | A | 21 | M | No | Non-ARDS | 2021.01.02 | 2021.01.09 |
| 6 | A | 33 | M | No | Non-ARDS | 2021.02.07 | 2021.02.12 |
| 7 | A | 53 | M | No | Non-ARDS | 2020.12.28 | 2021.01.05 |
| 8 | A | 61 | M | No | Non-ARDS | 2021.01.02 | 2021.01.23 |
| 9 | A | 58 | F | No | Non-ARDS | 2020.11.06 | 2021.11.06 |
| 10 | A | 41 | M | No | Non-ARDS | 2021.01.26 | 2021.02.07 |
| 11 | A | 46 | M | No | Non-ARDS | 2020.10.30 | 2020.11.06 |
| 12 | B | 67 | F | No | ARDS | 2020.12.20 | 2021.01.03 |
| 13 | B | 60 | F | No | ARDS | 2020.09.23 | 2020.10.09 |
| 14 | B | 62 | F | No | ARDS | NA | 2020.10.09 |
| 15 | B | 64 | F | No | ARDS | 2020.09.29 | 2020.10.09 |
| 16 | B | 67 | M | No | ARDS | NA | 2020.10.09 |
| 17 | B | 65 | M | No | ARDS | 2020.10.17 | 2020.10.22 |
| 18 | B | 48 | F | No | ARDS | 2020.10.30 | 2020.11.06 |
| 19 | B | 62 | M | No | ARDS | 2020.12.25 | 2020.12.29 |
| 20 | B | 68 | M | No | ARDS | 2020.11.27 | 2020.12.29 |
| 21 | B | 69 | M | No | ARDS | 2020.12.28 | 2021.01.09 |
| 22 | B | 70 | M | No | ARDS | 2020.11.02 | 2020.11.06 |
| 23 | B | 63 | F | No | ARDS | 2020.11.02 | 2020.11.06 |
| 24 | B | 64 | M | No | ARDS | 2020.12.16 | 2020.12.29 |
| 25 | B | 70 | M | No | ARDS | 2020.12.24 | 2020.12.29 |
| 26 | B | 67 | M | No | ARDS | 2020.12.11 | 2021.01.09 |
| 27 | C | 50 | M | Yes | ARDS | 2020.10.13 | 2020.10.22 |
| 28 | C | 76 | F | Yes | ARDS | 2020.11.14 | 2020.11.22 |
| 29 | C | 65 | F | Yes | ARDS | 2020.11.01 | 2020.11.06 |
| 30 | C | 46 | M | Yes | ARDS | 2020.09.29 | 2020.10.09 |
| 31 | C | 66 | F | Yes | ARDS | 2020.11.01 | 2020.11.06 |
| 32 | C | 53 | M | Yes | ARDS | 2020.10.03 | 2020.10.09 |
