# Supplementary material for: Impact of prophylactic hydroxychloroquine on ultrastructural impairment and cellular SARS-CoV-2 infection in different cells of bronchoalveolar lavage fluids of COVID-19 patients
Source: Sci Rep. 2023 Aug 5;13:12733. doi: 10.1038/s41598-023-39941-6 (PMC10404249; doi:10.1038/s41598-023-39941-6)
Supplement: Supplementary file 2 — Supplementary Table S2. [file 41598_2023_39941_MOESM2_ESM.docx]

**Supplementary Tables 2: Details of the number of images of each specific cell from the BALF of each patient’s subgroup used to analyze in this manuscript.**

| **Type of cells from BALF** | **Imaging methods used** | No. of the images taken in this study of specific cells from the BALF in each group of patients | | |
| --- | --- | --- | --- | --- |
|  |  | A | B | C |
| **Ciliated Epithelium** | **Pap** | 05 | 07 | 07 |
|  | **IF** | 12 | 10 | 14 |
|  | **SEM** | 04 | 07 | 06 |
|  | **TEM** | 04 | 07 | 06 |
| **Type II Pneumocytes** | **Pap** | 22 | 27 | 11 |
|  | **IF** | 18 | 10 | 09 |
|  | **SEM** | 6 | 5 | 3 |
|  | **TEM** | 13 | 10 | 8 |
| **Macrophages** | **Pap** | 12 | 17 | 13 |
|  | **IF** | 17 | 11 | 08 |
|  | **SEM** | 8 | 8 | 5 |
|  | **TEM** | 8 | 7 | 7 |
| **Neutrophils** | **Pap** | 13 | 13 | 11 |
|  | **IF** | 14 | 13 | 09 |
|  | **SEM** | 3 | 7 | 3 |
|  | **TEM** | 9 | 28 | 8 |
| **Enucleated Granulocytes** | **Pap** | 8 | 15 | 7 |
|  | **IF** | 07 | 04 | 05 |
|  | **SEM** | 5 | 6 | 7 |
|  | **TEM** | 2 | 5 | 8 |
