# Supplementary material for: Impact of prophylactic hydroxychloroquine on ultrastructural impairment and cellular SARS-CoV-2 infection in different cells of bronchoalveolar lavage fluids of COVID-19 patients
Source: Sci Rep. 2023 Aug 5;13:12733. doi: 10.1038/s41598-023-39941-6 (PMC10404249; doi:10.1038/s41598-023-39941-6)
Supplement: Supplementary file 3 — Supplementary Table S3. [file 41598_2023_39941_MOESM3_ESM.docx]

**Supplementary Table S3: Fluorescence intensity determination using Fiji software for the many cells in each patient subgroups**

| **Type of Cells** | **Sub-group** | **Above 60 Years (A)** | |
| --- | --- | --- | --- |
|  |  | **Fluor. Int of cells (number of cells examined)** | **Statis. Para.** |
| **Ciliated Epithelium** | **A** | 21.48; 21.64; 13.359; 13.114; 15.255; 19.968; 13.013; 22.463; 15.127; 13.483; 22.01; 21.65; | N=12  M=17.71  SD=4.10 |
|  | **B** | 27.121; 22.390; 14.684; 16.184; 16.895; 20.231; 21.318; 24.660; 31.797; 24.887 | N=10  M=22.02  SD=5.32 |
|  | **C** | 10.604; 12.358; 10.526; 11.042; 14.529; 10.080; 10.099; 10.317; 12.765; 16.022; 13.117; 18.817; 9.489; 10.651 | N=14  M=12.17  SD= 2.69 |
| **Type II pneumocytes** | **A** | 16.203; 15.959; 17.116; 16.091; 15.282; 19.09; 15.798; 19.809; 21.97; 17.746; 24.531; 29.248; 17.732; 22.807; 21.557; 19.360; 18.846; 22.415 | N=18  M=19.53  SD=3.67 |
|  | **B** | 21.199; 19.695; 19.042; 17.766; 22.219; 18.189; 21.96; 23.51; 21.122; 15.226 | N=10  M=19.99  SD=2.49 |
|  | **C** | 11.162; 10.944; 11.537; 12.427; 10.120; 16.059; 11.118; 11.279; 10.961; | N=09  M=11.73  SD=1.73 |
| **Macrophage** | **A** | 12.511; 9.551; 13.514; 11.018; 15.288; 10.744; 16.481; 10.955; 14.819; 12.536; 18.771; 11.132; 18.034; 14.387; 19.360; 17.904; 18.964 | N=17  M=14.47  SD=3.30 |
|  | **B** | 18.092; 15.999; 13.959; 14.947; 11.976; 18.453; 14.183; 12.784; 14.914; 19.653; 13.268 | N=11  M=15.29  SD=2.50 |
|  | **C** | 13.785; 17.575; 19.865; 14.856; 14.717; 15.903; 12.714; 13.270; | N=8  M=15.33  SD=2.39 |
| **Neutrophil** | **A** | 13.24; 13.347; 11.333; 10.35; 19.797; 12.556; 12.444; 17.934; 16.820; 20.005; 20.980; 12.589; 20.387; 15.887 | N=14  M=15.55  SD=3.73 |
|  | **B** | 10.814; 19.365; 14.921; 15.297; 14.125; 17.789; 16.85; 15.262; 17.857; 13.628; 18.074; 17.151; 10.51 | N=13  M=15.51  SD=2.73 |
|  | **C** | 14.484; 13.21; 19.946; 17.656; 14.412; 10.67; 17.60; 13.899; 16.845; | N=09  M=15.41  SD=2.83 |
| **Anucleated Granulocytes** | **A** | 19.321; 23.704; 28.520; 23.381; 22.123; 22.217; 26.889 | N=07  M=23.74  SD=3.09 |
|  | **B** | 19.310; 24.42; 25.337; 26.118; | N=4  M=23.79  SD=3.07 |
|  | **C** | 23.771; 27.660; 22.861; 24.083; 19.101; | N=5  M=23.49  SD=3.06 |
