# Supplementary figures and images for: Impact of prophylactic hydroxychloroquine on ultrastructural impairment and cellular SARS-CoV-2 infection in different cells of bronchoalveolar lavage fluids of COVID-19 patients
Source: Sci Rep. 2023 Aug 5;13:12733. doi: 10.1038/s41598-023-39941-6 (PMC10404249; doi:10.1038/s41598-023-39941-6)

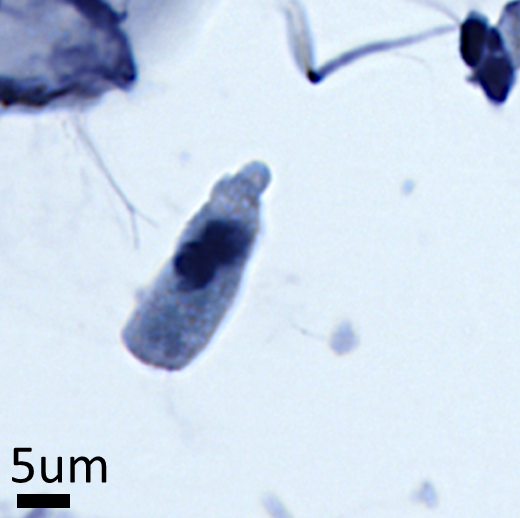

Supplement: Supplementary file 4 — Supplementary Information 1. [file 41598_2023_39941_MOESM4_ESM.zip › Supplementry File for Raw data/01. Fig. 2A (PAP).png]

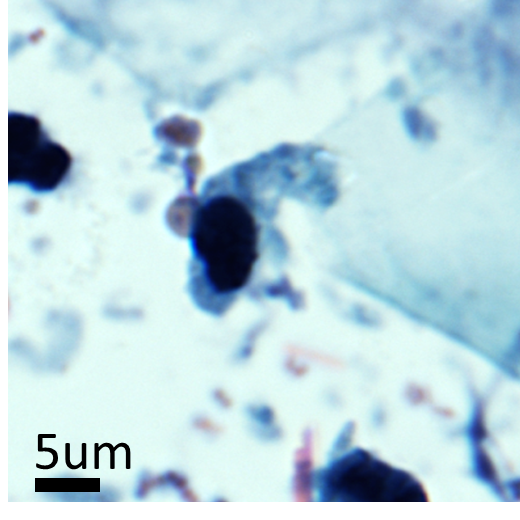

Supplement: Supplementary file 4 — Supplementary Information 1. [file 41598_2023_39941_MOESM4_ESM.zip › Supplementry File for Raw data/17. Fig. 3B (PAP).png]

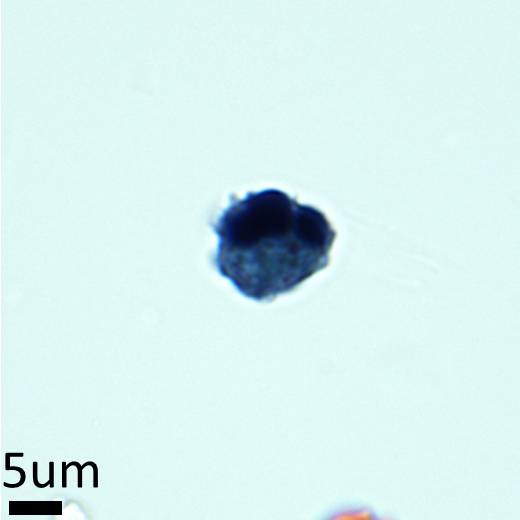

Supplement: Supplementary file 4 — Supplementary Information 1. [file 41598_2023_39941_MOESM4_ESM.zip › Supplementry File for Raw data/37. Fig. 5A (PAP).png]

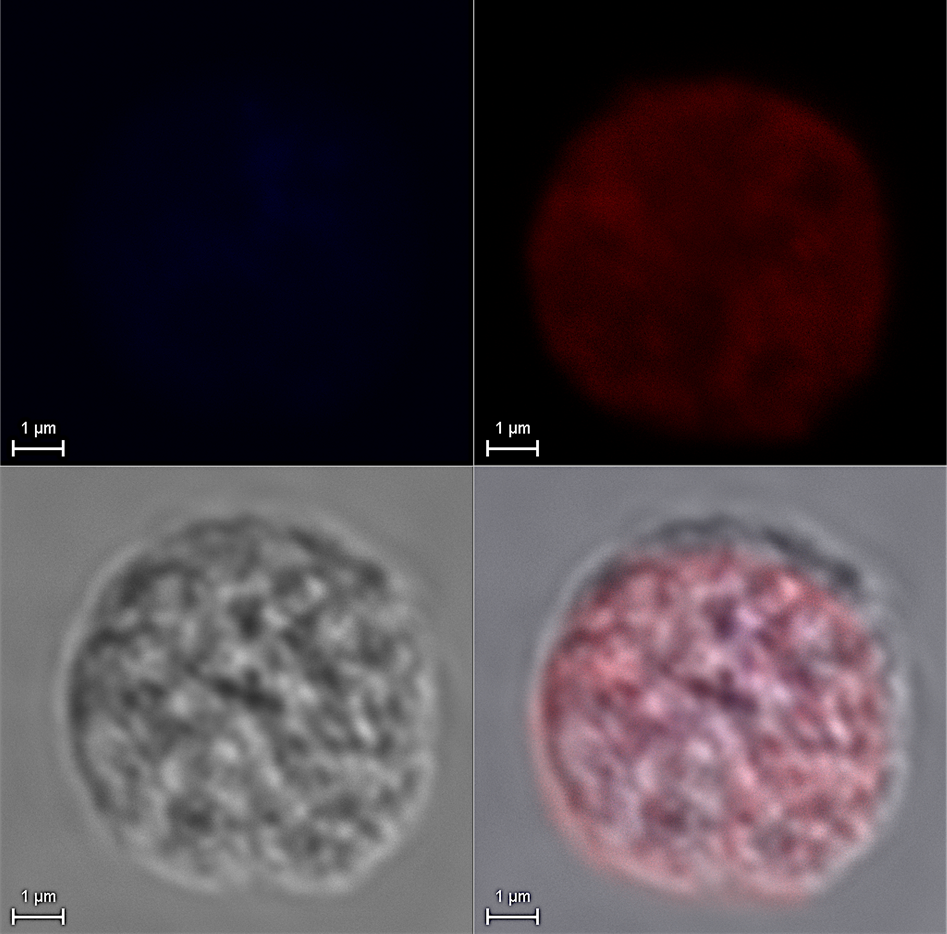

Supplement: Supplementary file 4 — Supplementary Information 1. [file 41598_2023_39941_MOESM4_ESM.zip › Supplementry File for Raw data/54. Fig. 6B (IF).png]

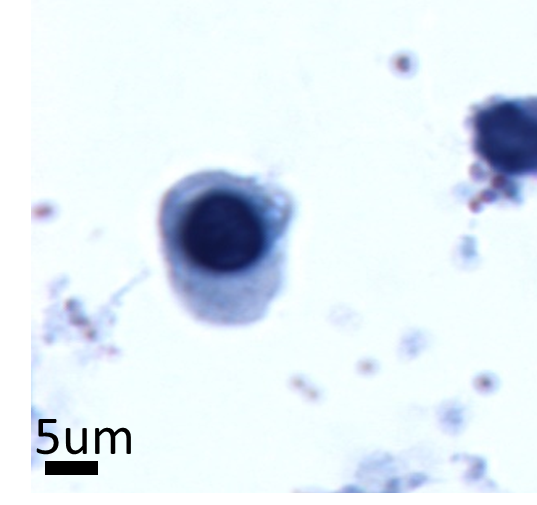

Supplement: Supplementary file 4 — Supplementary Information 1. [file 41598_2023_39941_MOESM4_ESM.zip › Supplementry File for Raw data/21. Fig. 3C (PAP).png]

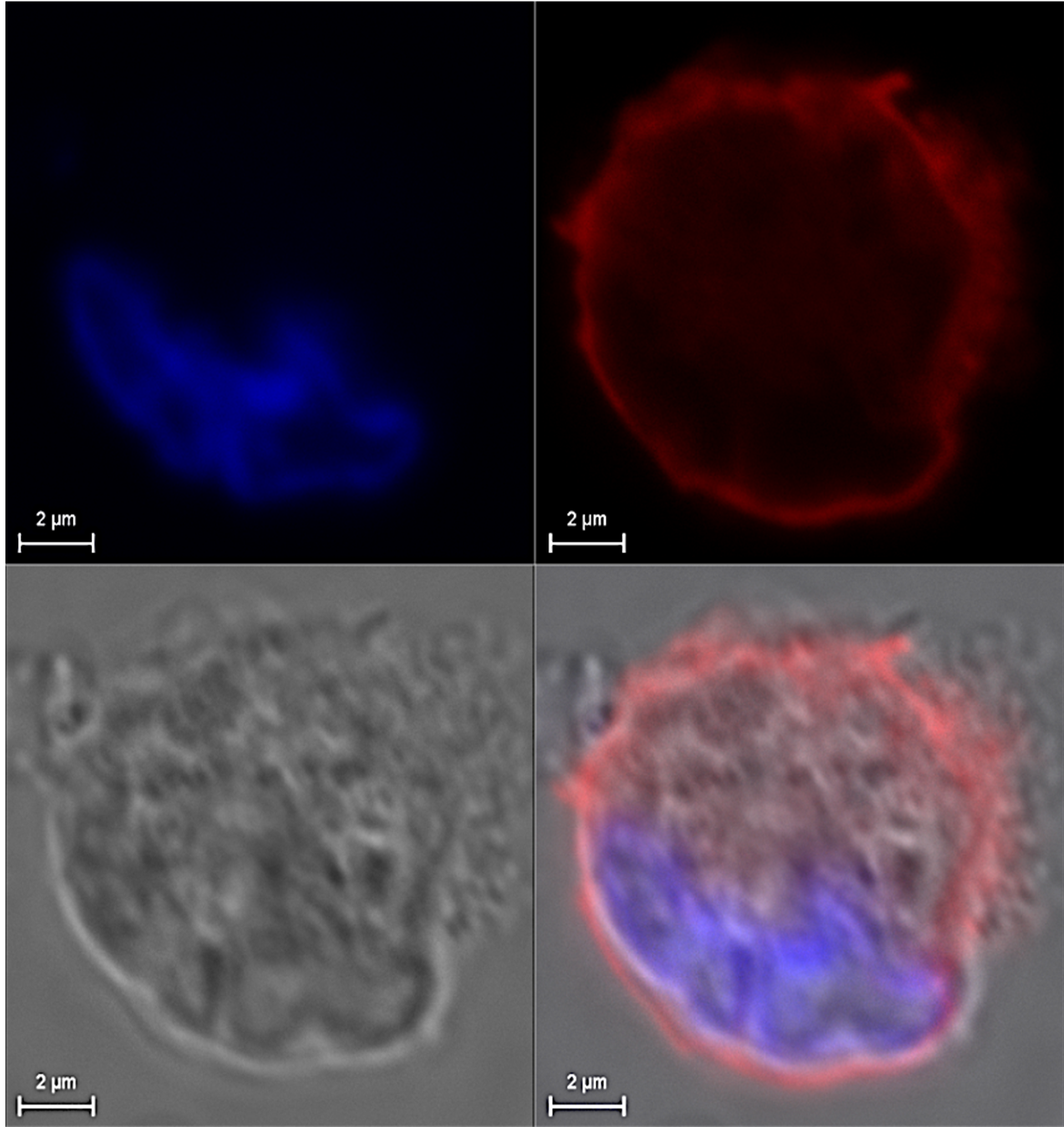

Supplement: Supplementary file 4 — Supplementary Information 1. [file 41598_2023_39941_MOESM4_ESM.zip › Supplementry File for Raw data/46. Fig. 5C (IF).png]

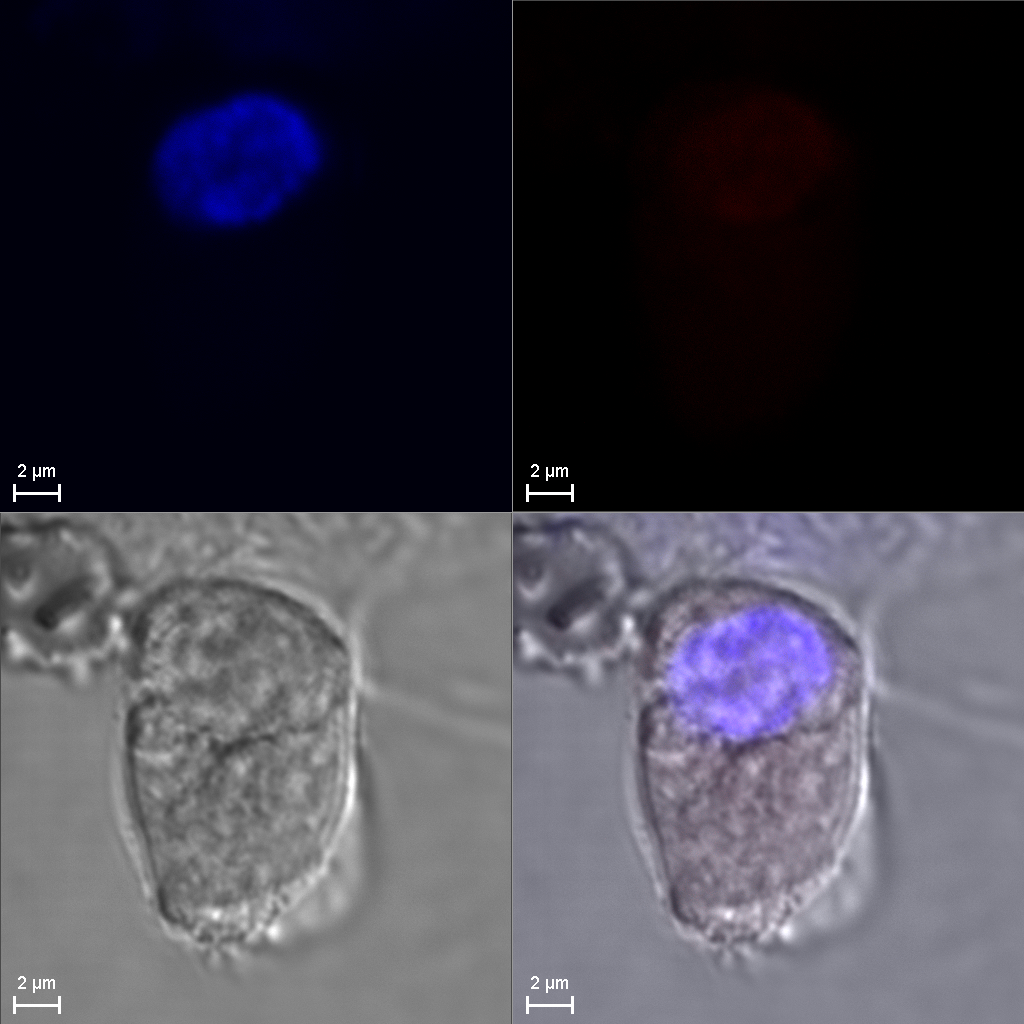

Supplement: Supplementary file 4 — Supplementary Information 1. [file 41598_2023_39941_MOESM4_ESM.zip › Supplementry File for Raw data/22. Fig. 3C (IF).tif]

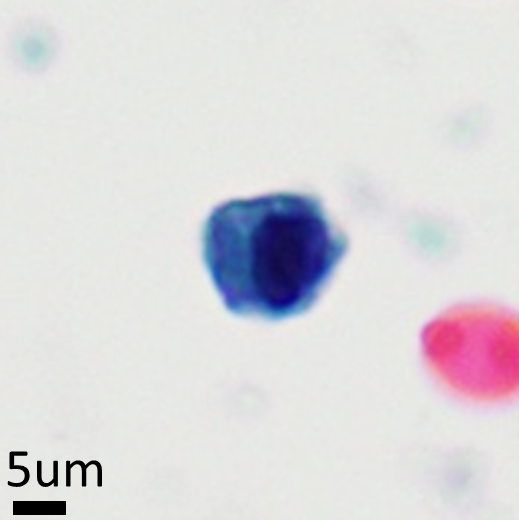

Supplement: Supplementary file 4 — Supplementary Information 1. [file 41598_2023_39941_MOESM4_ESM.zip › Supplementry File for Raw data/29. Fig. 4B (PAP).png]

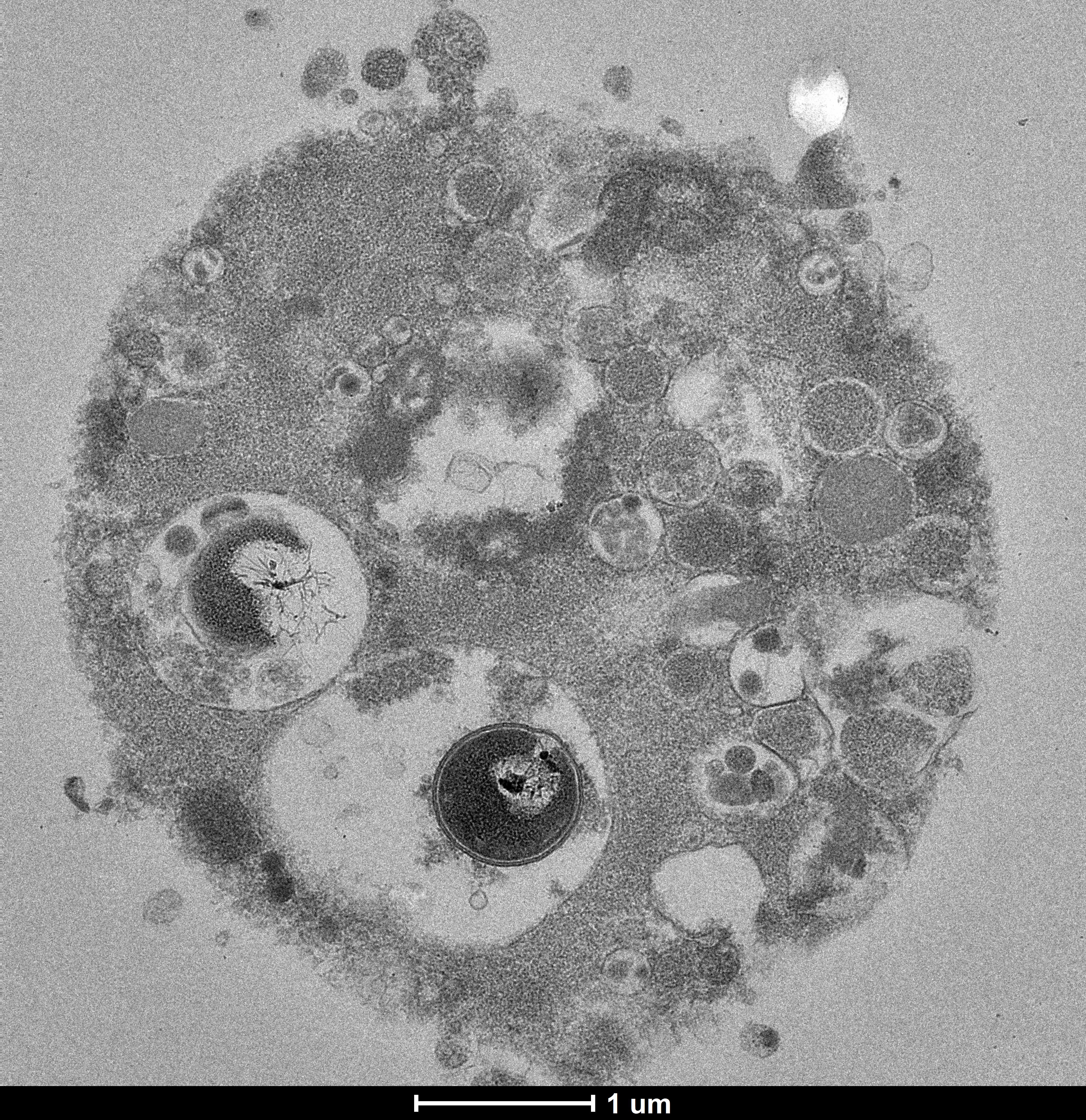

Supplement: Supplementary file 4 — Supplementary Information 1. [file 41598_2023_39941_MOESM4_ESM.zip › Supplementry File for Raw data/52. Fig. 6A (TEM).jpg]

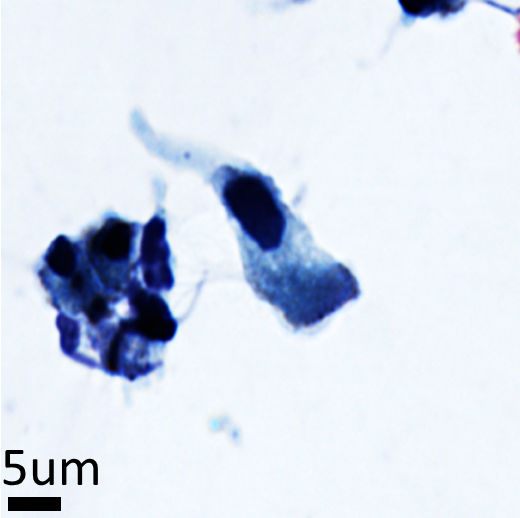

Supplement: Supplementary file 4 — Supplementary Information 1. [file 41598_2023_39941_MOESM4_ESM.zip › Supplementry File for Raw data/05. Fig. 2B (PAP).png]

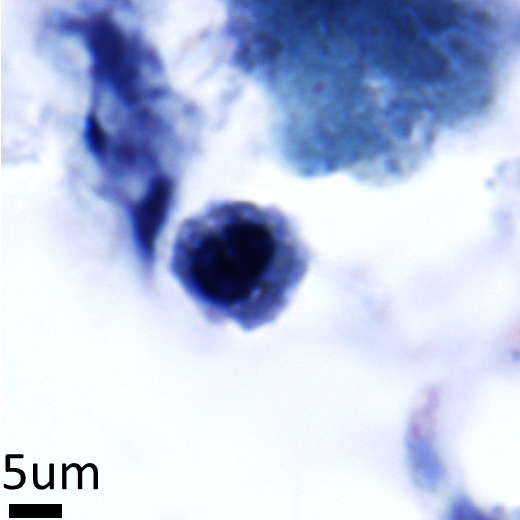

Supplement: Supplementary file 4 — Supplementary Information 1. [file 41598_2023_39941_MOESM4_ESM.zip › Supplementry File for Raw data/25. Fig. 4A (PAP).png]

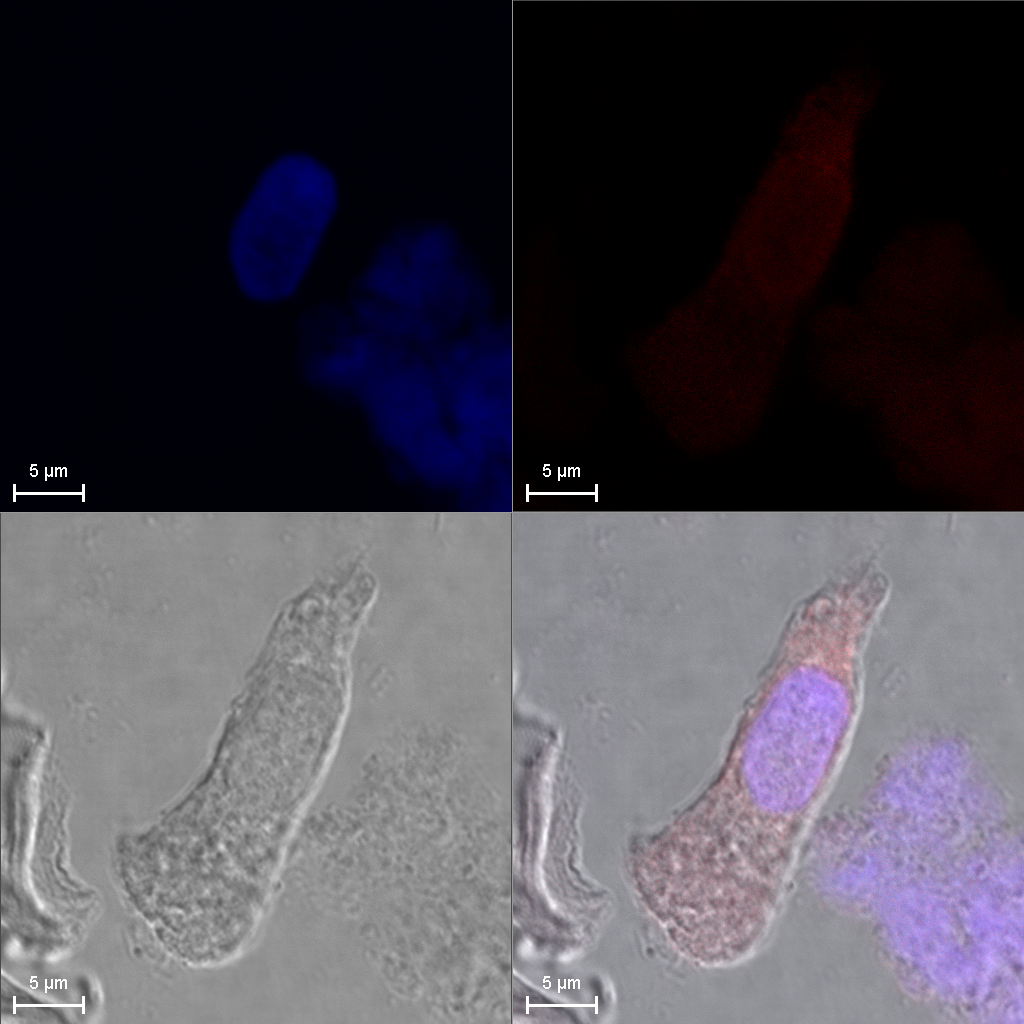

Supplement: Supplementary file 4 — Supplementary Information 1. [file 41598_2023_39941_MOESM4_ESM.zip › Supplementry File for Raw data/02. Fig. 2A (IF).tif]

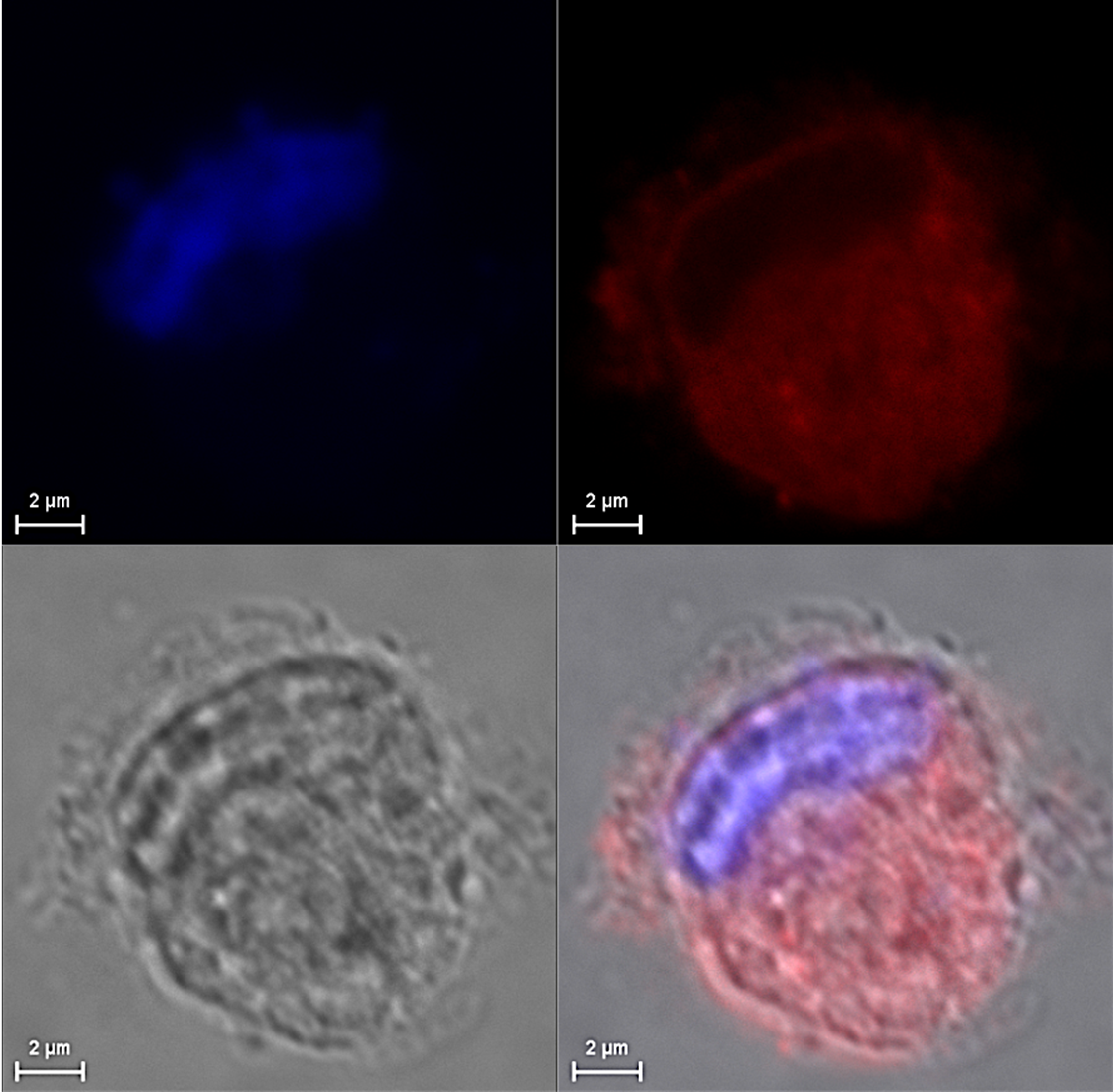

Supplement: Supplementary file 4 — Supplementary Information 1. [file 41598_2023_39941_MOESM4_ESM.zip › Supplementry File for Raw data/42. Fig. 5B (IF).png]

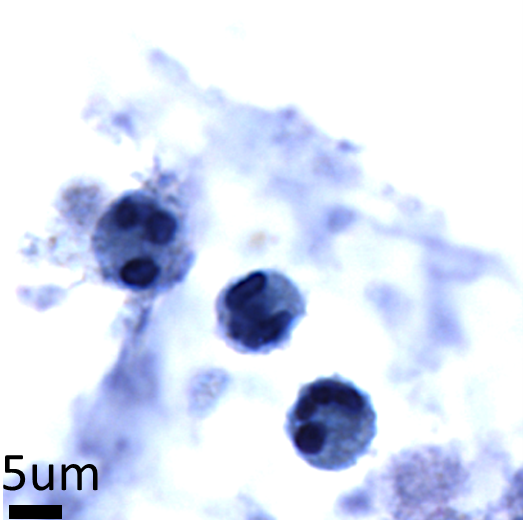

Supplement: Supplementary file 4 — Supplementary Information 1. [file 41598_2023_39941_MOESM4_ESM.zip › Supplementry File for Raw data/45. Fig. 5C (PAP).png]

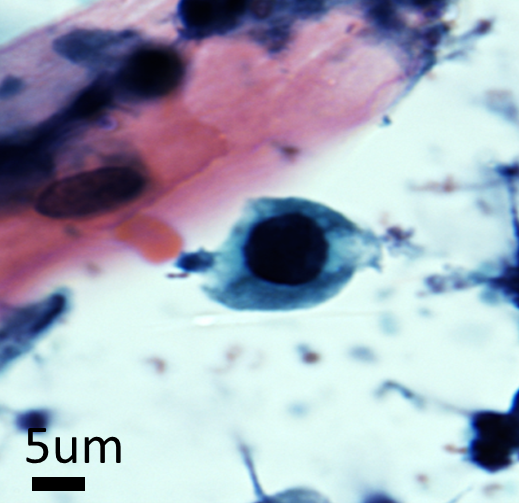

Supplement: Supplementary file 4 — Supplementary Information 1. [file 41598_2023_39941_MOESM4_ESM.zip › Supplementry File for Raw data/13. Fig. 3A (PAP).png]

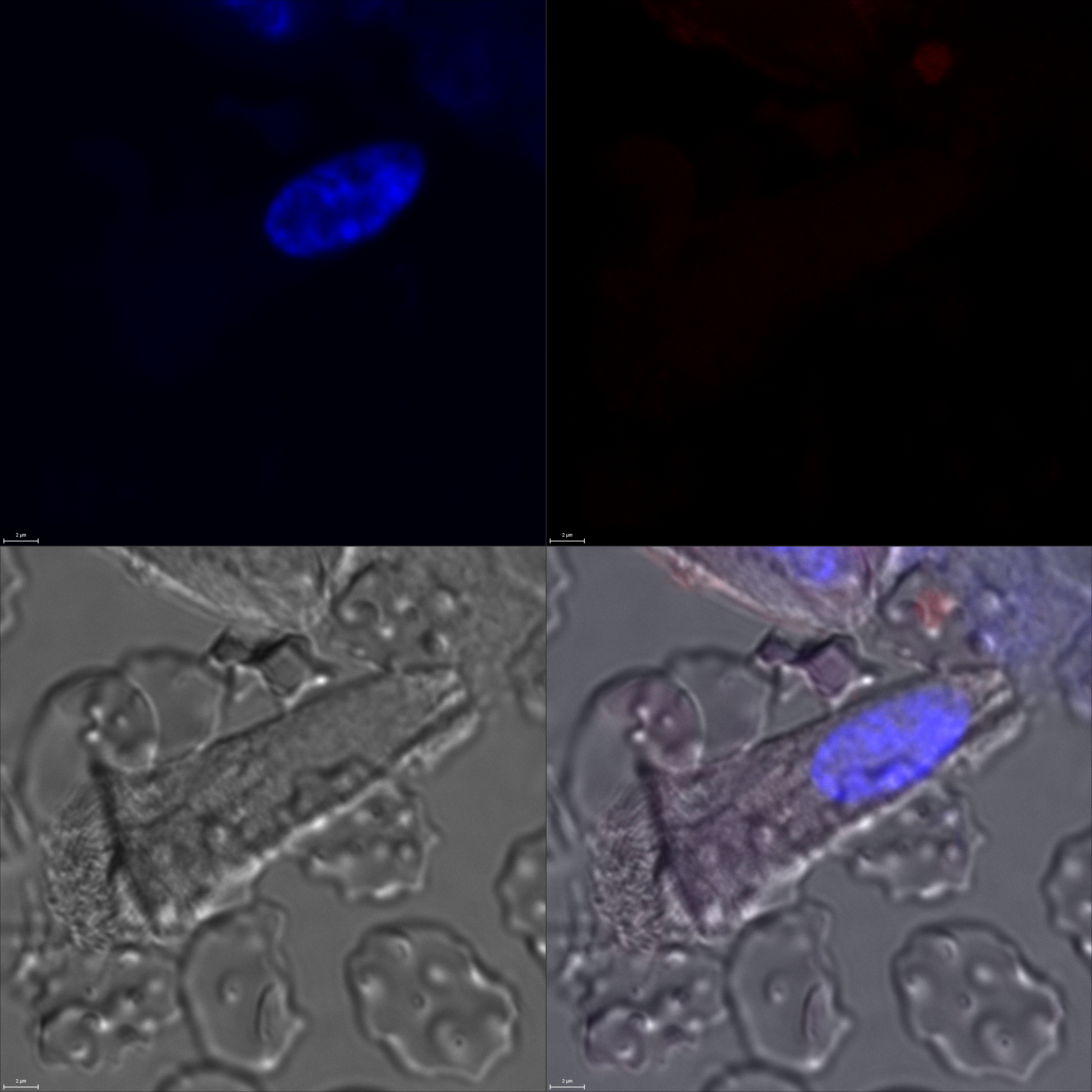

Supplement: Supplementary file 4 — Supplementary Information 1. [file 41598_2023_39941_MOESM4_ESM.zip › Supplementry File for Raw data/10. Fig. 2C (IF).tif]

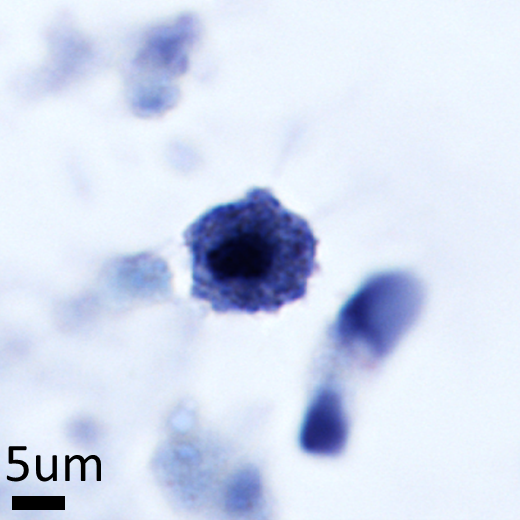

Supplement: Supplementary file 4 — Supplementary Information 1. [file 41598_2023_39941_MOESM4_ESM.zip › Supplementry File for Raw data/33. Fig. 4C (PAP).png]

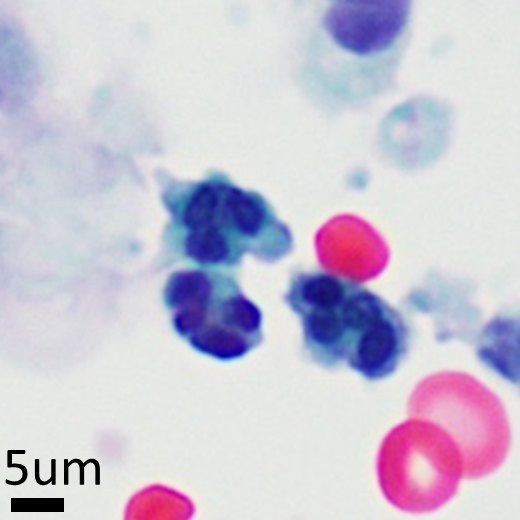

Supplement: Supplementary file 4 — Supplementary Information 1. [file 41598_2023_39941_MOESM4_ESM.zip › Supplementry File for Raw data/41. Fig. 5B (PAP).png]

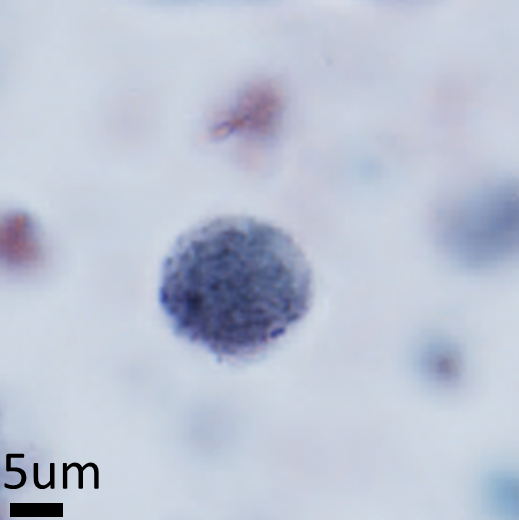

Supplement: Supplementary file 4 — Supplementary Information 1. [file 41598_2023_39941_MOESM4_ESM.zip › Supplementry File for Raw data/53. Fig. 6B (PAP).png]

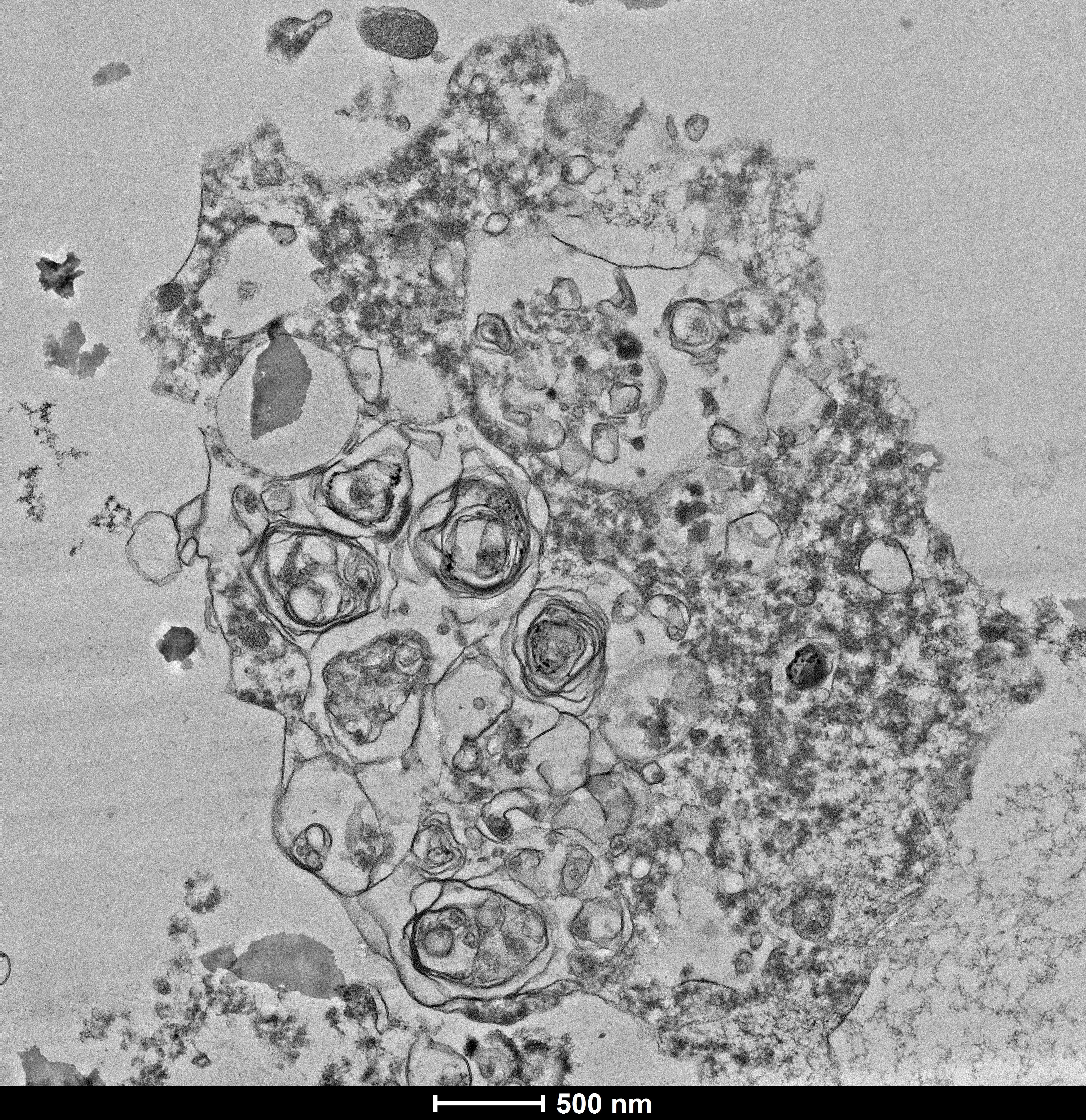

Supplement: Supplementary file 4 — Supplementary Information 1. [file 41598_2023_39941_MOESM4_ESM.zip › Supplementry File for Raw data/24. Fig. 3C (TEM).jpg]

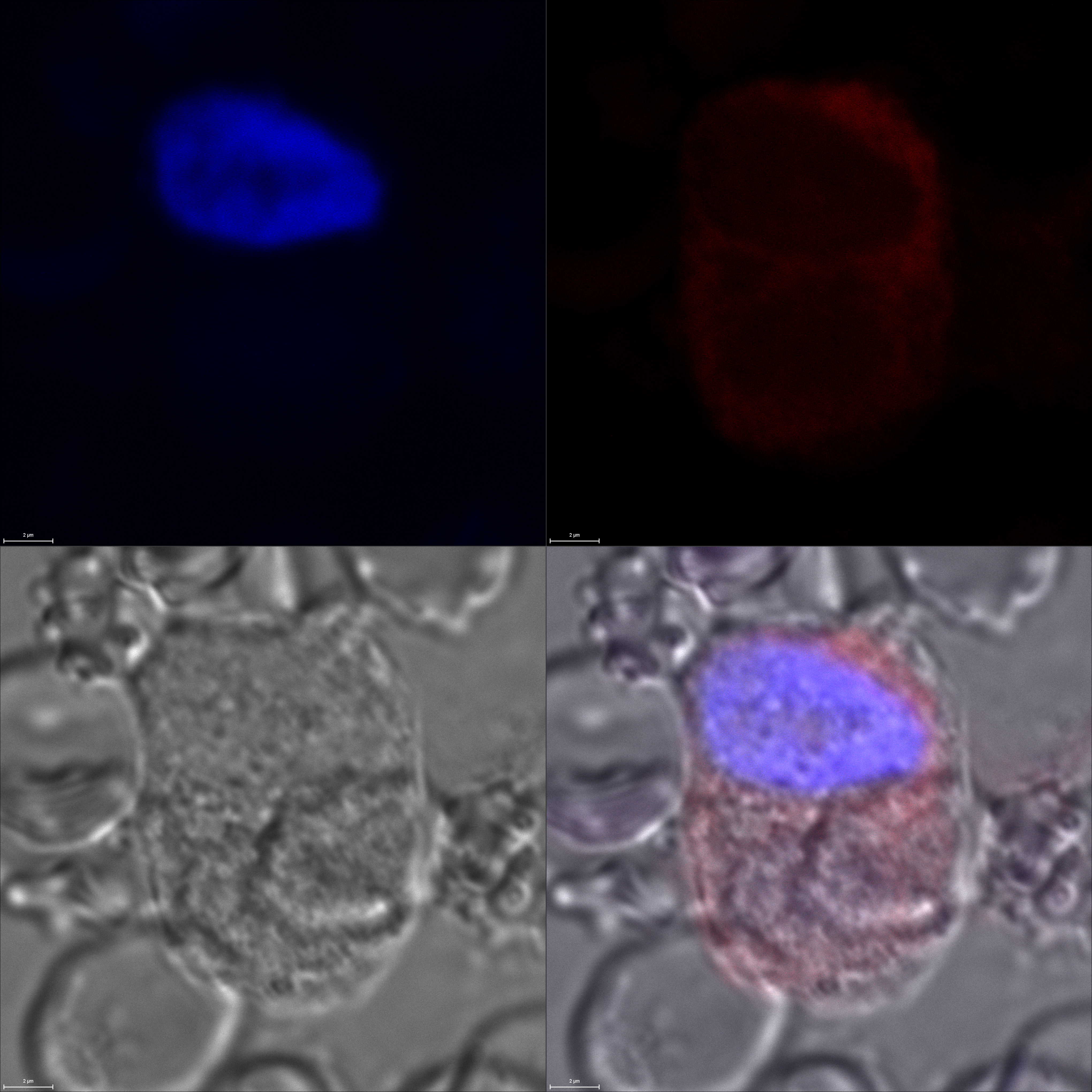

Supplement: Supplementary file 4 — Supplementary Information 1. [file 41598_2023_39941_MOESM4_ESM.zip › Supplementry File for Raw data/14. Fig. 3A (IF).tif]

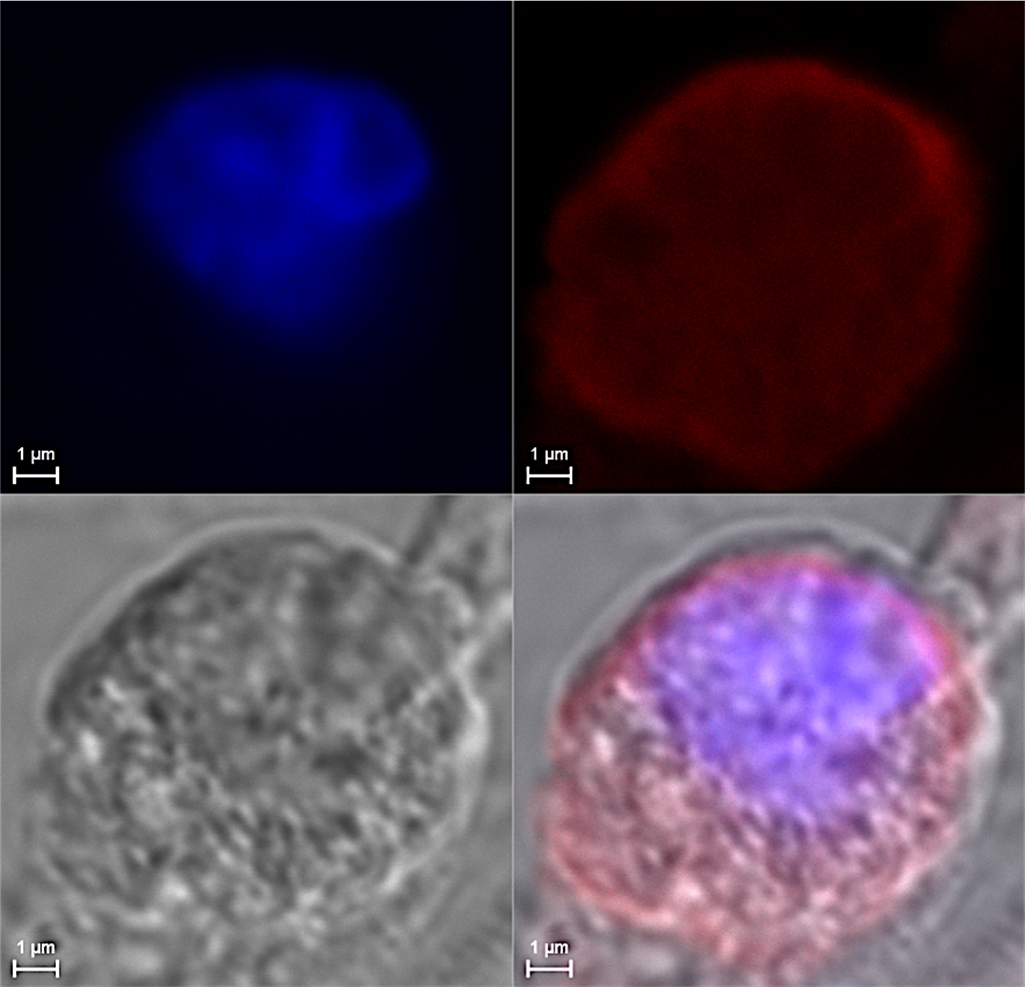

Supplement: Supplementary file 4 — Supplementary Information 1. [file 41598_2023_39941_MOESM4_ESM.zip › Supplementry File for Raw data/26. Fig. 4A (IF).png]

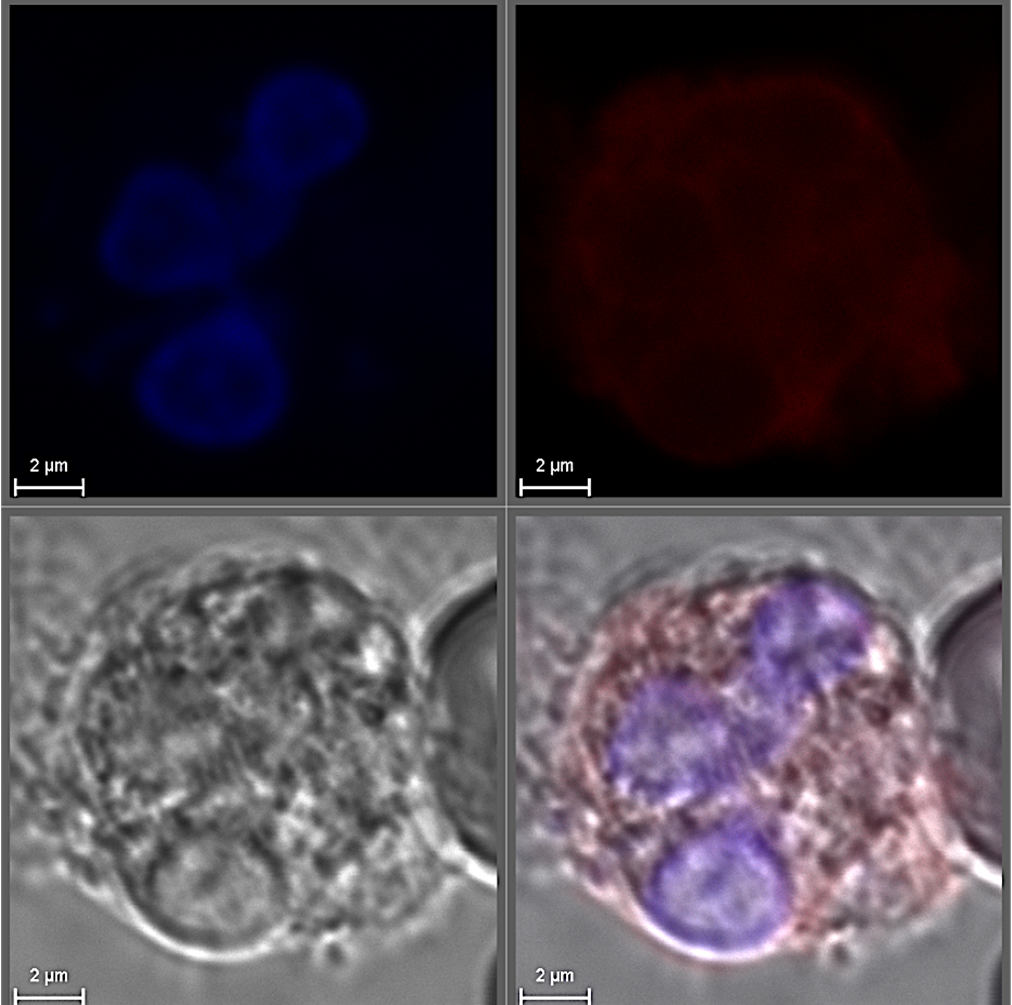

Supplement: Supplementary file 4 — Supplementary Information 1. [file 41598_2023_39941_MOESM4_ESM.zip › Supplementry File for Raw data/38. Fig. 5A (IF).png]

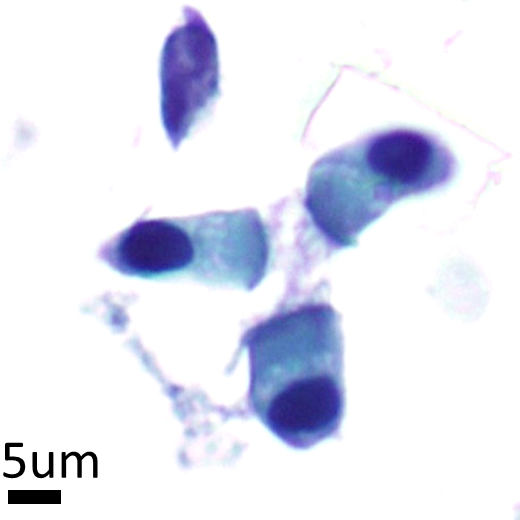

Supplement: Supplementary file 4 — Supplementary Information 1. [file 41598_2023_39941_MOESM4_ESM.zip › Supplementry File for Raw data/09. Fig. 2C (PAP).png]

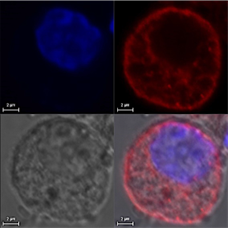

Supplement: Supplementary file 4 — Supplementary Information 1. [file 41598_2023_39941_MOESM4_ESM.zip › Supplementry File for Raw data/34. Fig. 4C (IF).png]

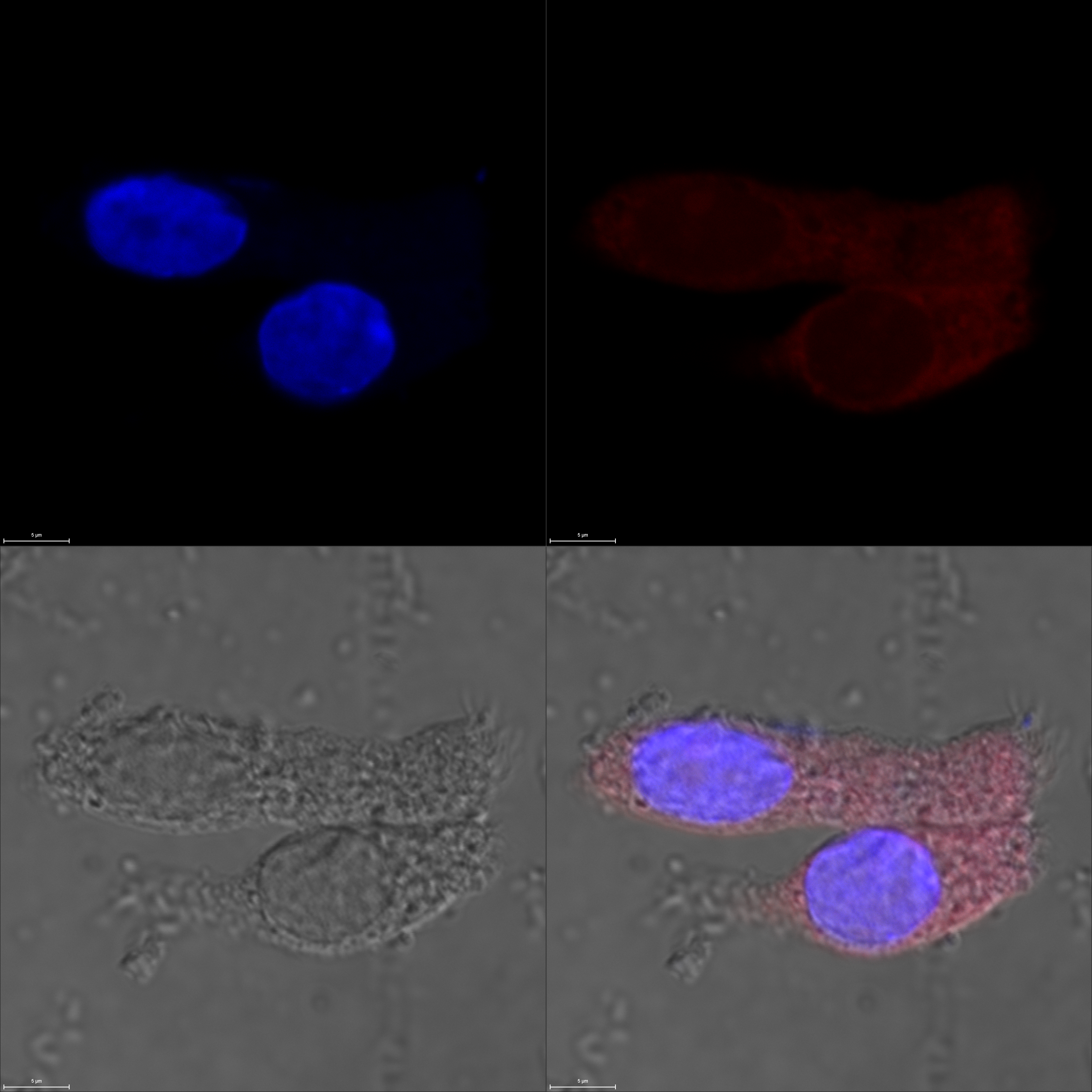

Supplement: Supplementary file 4 — Supplementary Information 1. [file 41598_2023_39941_MOESM4_ESM.zip › Supplementry File for Raw data/06. Fig. 2B (IF).tif]

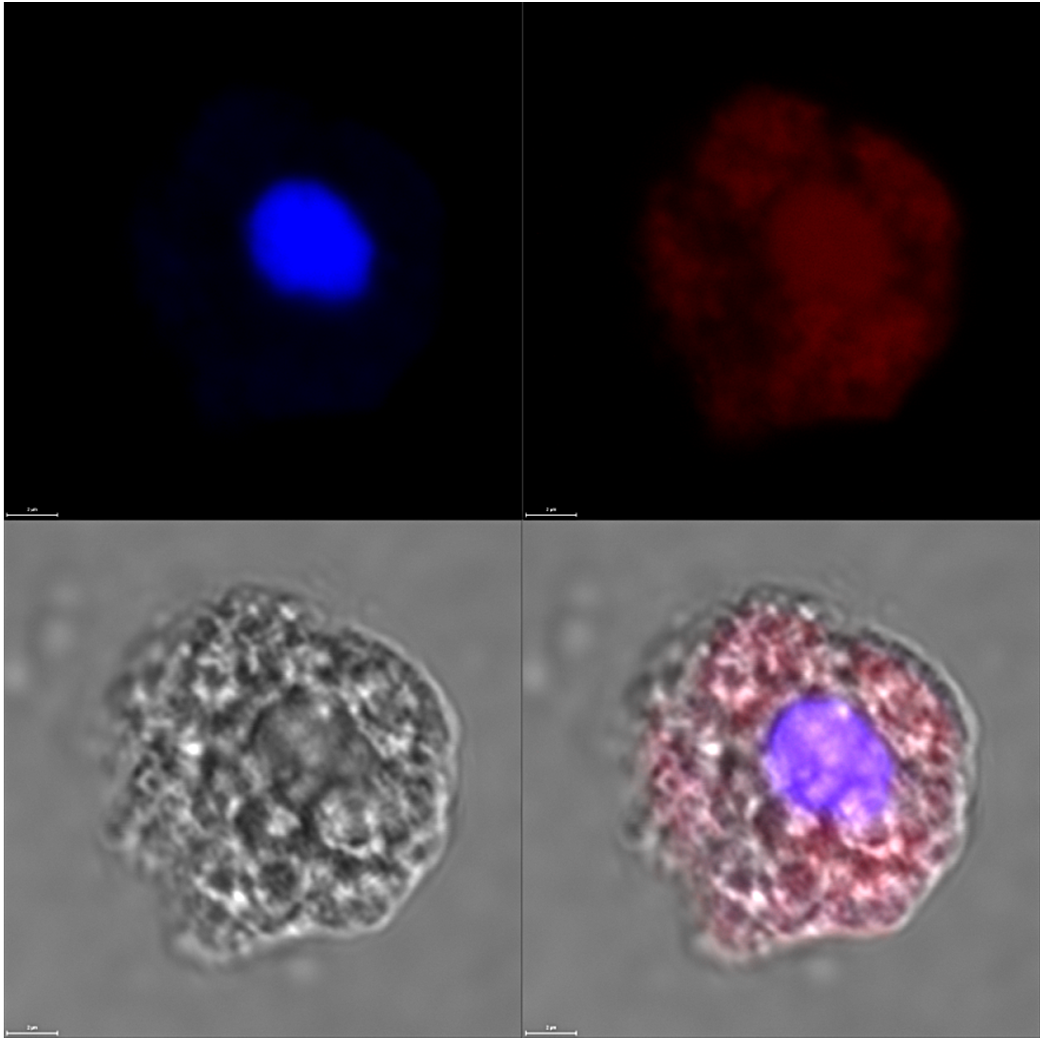

Supplement: Supplementary file 4 — Supplementary Information 1. [file 41598_2023_39941_MOESM4_ESM.zip › Supplementry File for Raw data/30. Fig. 4B (IF).png]

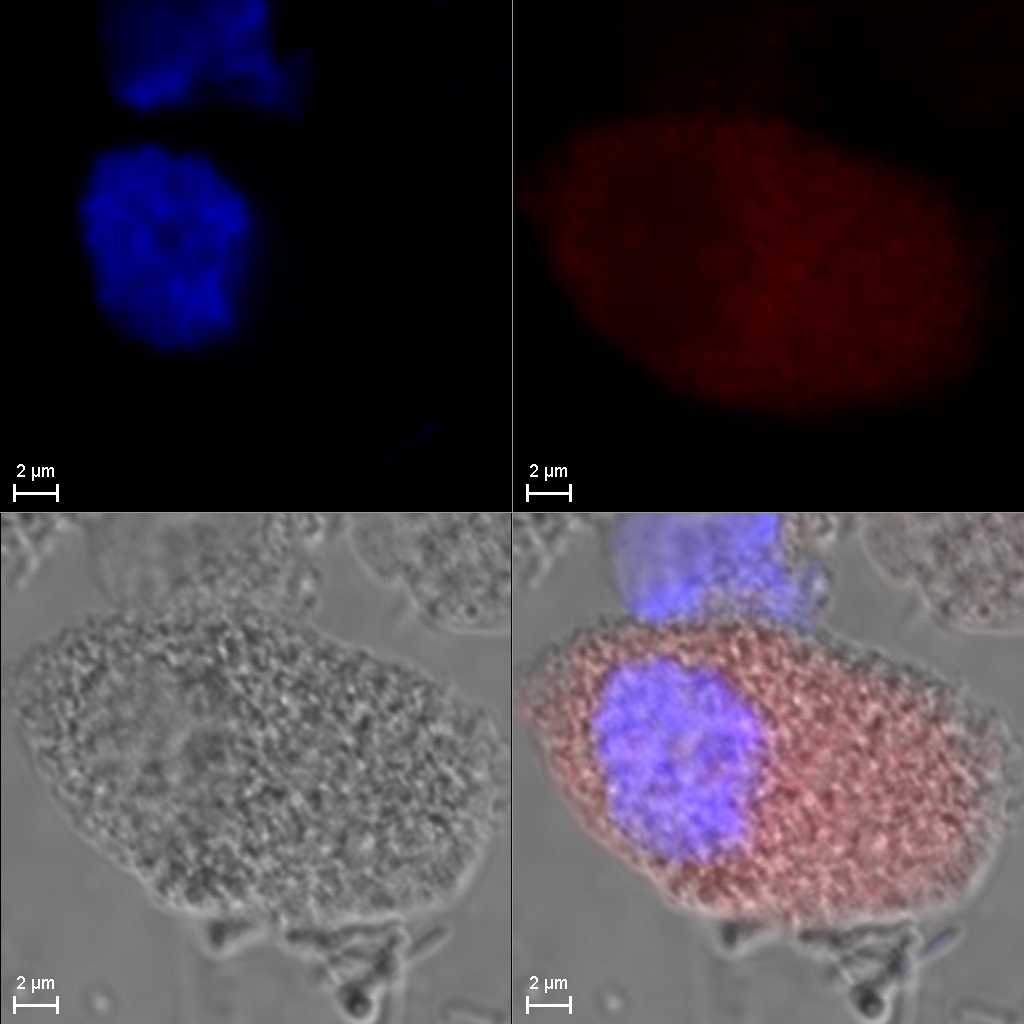

Supplement: Supplementary file 4 — Supplementary Information 1. [file 41598_2023_39941_MOESM4_ESM.zip › Supplementry File for Raw data/18. Fig. 3B (IF).tif]

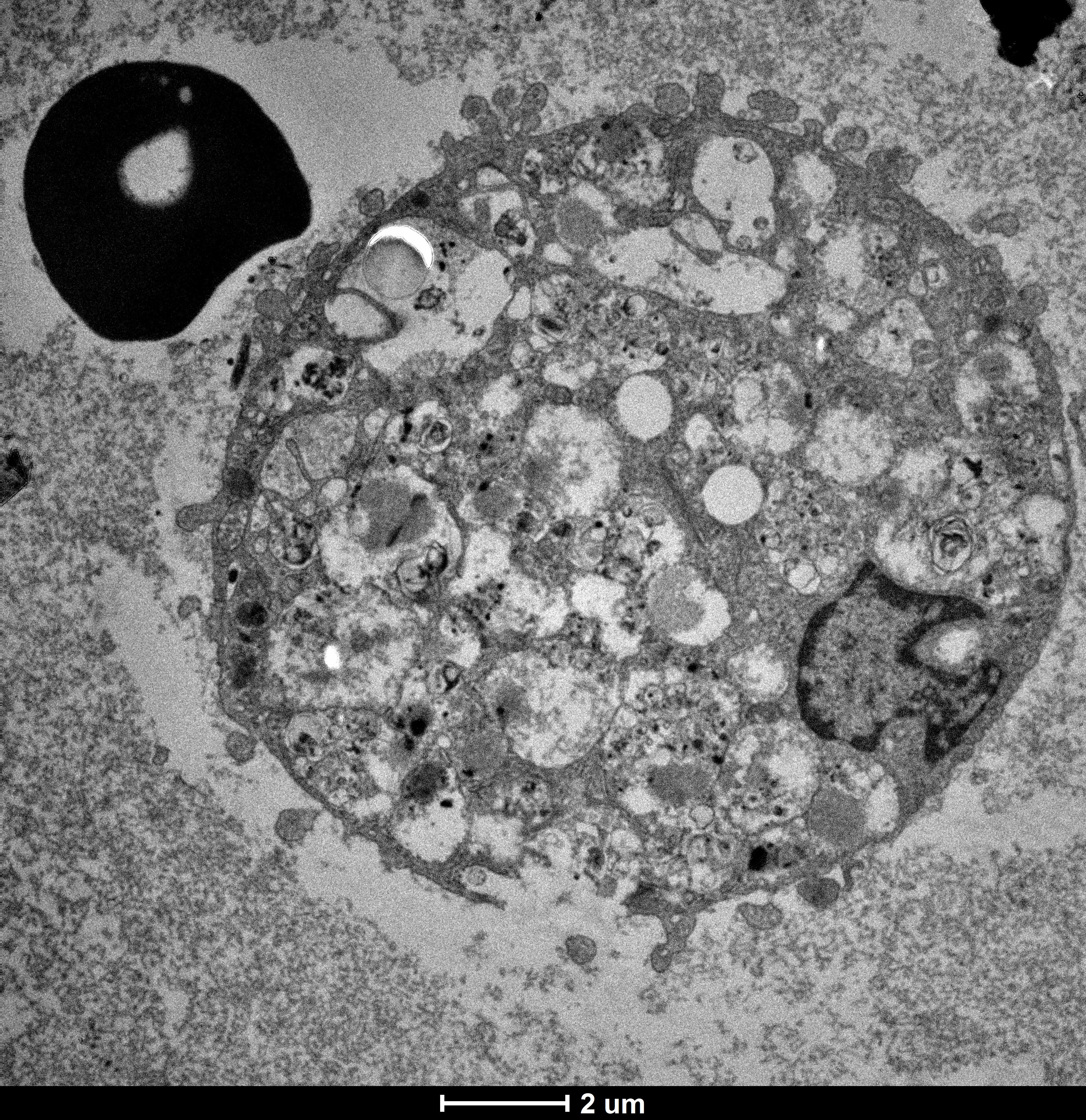

Supplement: Supplementary file 4 — Supplementary Information 1. [file 41598_2023_39941_MOESM4_ESM.zip › Supplementry File for Raw data/16. Fig. 3A (TEM).jpg]

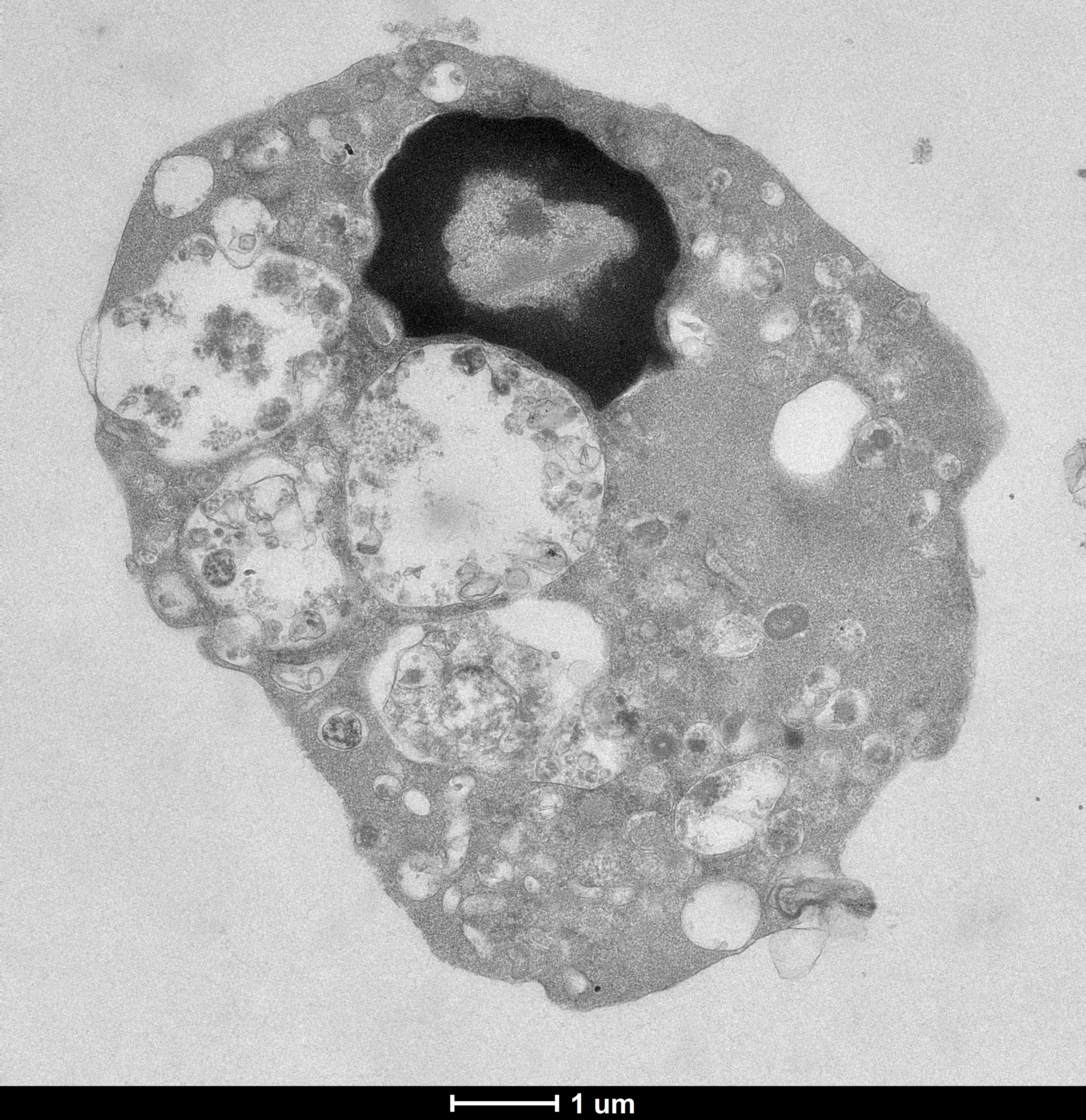

Supplement: Supplementary file 4 — Supplementary Information 1. [file 41598_2023_39941_MOESM4_ESM.zip › Supplementry File for Raw data/32. Fig. 4B (TEM).jpg]

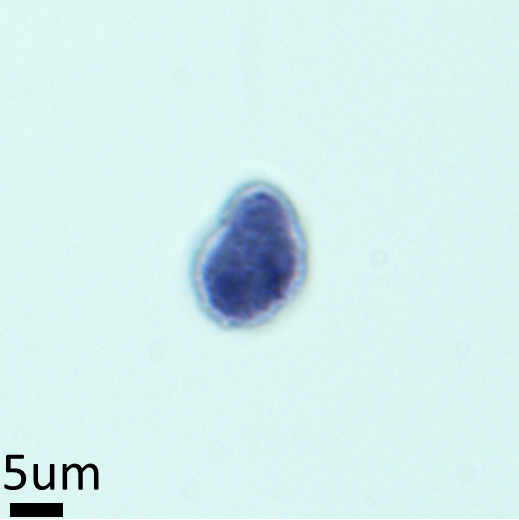

Supplement: Supplementary file 4 — Supplementary Information 1. [file 41598_2023_39941_MOESM4_ESM.zip › Supplementry File for Raw data/49. Fig. 6A (PAP).png]
